# Unprecedented generation of 3D heterostructures by mechanochemical disassembly and re-ordering of incommensurate metal chalcogenides

**DOI:** 10.1038/s41467-020-16672-0

**Published:** 2020-06-12

**Authors:** Oleksandr Dolotko, Ihor Z. Hlova, Arjun K. Pathak, Yaroslav Mudryk, Vitalij K. Pecharsky, Prashant Singh, Duane D. Johnson, Brett W. Boote, Jingzhe Li, Emily A. Smith, Scott L. Carnahan, Aaron J. Rossini, Lin Zhou, Ely M. Eastman, Viktor P. Balema

**Affiliations:** 1 Ames Laboratory of US Department of Energy, Iowa State University, Ames, IA 50011-2416 USA; 2 Department of Physics, SUNY Buffalo State, Buffalo, NY 14222 USA; 3 Department of Materials Science and Engineering, Iowa State University, Ames, IA 50011-1096 USA; 4 Department of Chemistry, Iowa State University, Ames, IA 50011-1021 USA; 5 Reed College, Portland, OR 97202-8199 USA

**Keywords:** Synthesis and processing, Nanoscience and technology

## Abstract

Three-dimensional heterostructures are usually created either by assembling two-dimensional building blocks into hierarchical architectures or using stepwise chemical processes that sequentially deposit individual monolayers. Both approaches suffer from a number of issues, including lack of suitable precursors, limited reproducibility, and poor scalability of the preparation protocols. Therefore, development of alternative methods that enable preparation of heterostructured materials is desired. We create heterostructures with incommensurate arrangements of well-defined building blocks using a synthetic approach that comprises mechanical disassembly and simultaneous reordering of layered transition-metal dichalcogenides, MX_2_, and non-layered monochalcogenides, REX, where M = Ta, Nb, RE = Sm, La, and X = S, Se. We show that the discovered solid-state processes are rooted in stochastic mechanochemical transformations directed by electronic interaction between chemically and structurally dissimilar solids toward atomic-scale ordering, and offer an alternative to conventional heterostructuring. Details of composition–structure–properties relationships in the studied materials are also highlighted.

## Abstract (web summary)

3D heterostructures offer properties that are inaccessible in bulk single-phase solids, but synthetic approaches are limited. The authors use mechanochemical reshuffling of binary precursors and subsequent annealing to design structurally aligned misfit heterostructures with well-defined atomic arrangements.

## Introduction

Three-dimensional (3D) heterostructures incorporating layered van der Waals (vdW)-type metal chalcogenides into their frameworks represent a new generation of hierarchical systems with unique properties inaccessible in bulk single-phase solids^1–4^. Typically, 3D heterostructures are created in two fundamentally different ways. The first, known as top-down synthesis, employs two-dimensional (2D) building blocks to assemble 3D-hierarchical architectures using an appropriate additive manufacturing technique. In this case, 2D precursors are prepared by exfoliating bulk vdW materials, such as graphite, boron nitride, black phosphorus, or transition-metal dichalcogenides (TMDCs)^1,2^. The second approach, broadly defined as bottom-up synthesis, produces hierarchical 3D hetero-assemblies using stepwise chemical reactions between pure elements or small molecules that deposit individual 2D monolayers on top of each other. Often, the deposition is carried out from a gas phase^5–8^ and involves transport agents^3,4^. While technically viable, both approaches suffer from a number of issues, including lack of suitable 2D precursors, insufficient process control and reproducibility, as well as poor scalability of the preparation protocols, which limit their practical applications. Hence, development of alternative methods to enable efficient preparation of 3D heterostructures has been identified as a major challenge for materials synthesis research^1,9^.

Recently, we discovered a mechanically facilitated “reshuffling” of binary TMDCs into 3D heterostructures^10^, where slabs of different metal dichalcogenides form layered assemblies that are tied together by weak vdW forces. The unexpected formation of hierarchical materials during mechanical milling, which is often associated with disintegration and amorphization of solids, raises an intriguing question about the ability of the uncovered phenomenon to deliver ordered 3D heterostructures, and its potential as a scalable and convenient alternative to the conventional top-down and bottom-up syntheses.

**Fig. 1:**
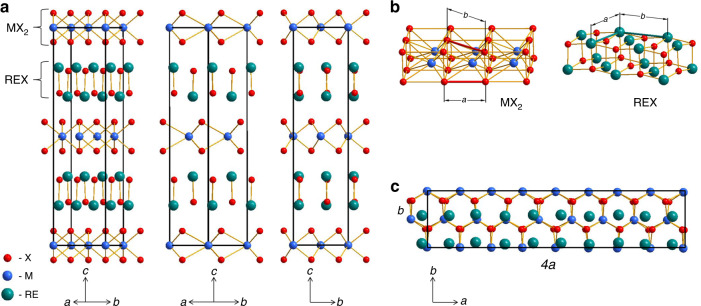
Schematic illustration of 3D heterostructures. Schematic of (REX)_1 + *x*_(MX_2_), where M = transition metal, for example, Nb or Ta, RE = rare-earth metal, for example, La or Sm, and X = S or Se. **a** The three different projections shown can be observed by STEM imaging. **b** Perspective views of MX_2_ and REX building blocks. **c** Projection of the heterostructure shown in **a** along [0 0 1] direction highlighting incommensurability along the *a*-axis with chalcogen atoms in the REX fragments omitted for clarity.

Among known 3D heterostructures, materials combining alternating layers of vdW TMDCs (MX_2_, M = transition metal; X = S or Se) and slabs of ionic rare-earth monochalcogenides (REX, RE = rare-earth metal) in the same superlattice are of particular interest. Such materials are often described as (REX)_1 + *x*_(MX_2_), where 1 + *x* = (4*a*_2_/2*a*_1_) and *a* parameters represent the lattice parameters of the individual REX and MX_2_ compounds^11^. Periodicities of the MX_2_ and REX building blocks in (REX)_1 + *x*_(MX_2_) are incommensurate along the *a*-axis, but they are commensurate along two other axes, *b* and *c* (Fig. 1). For example, the material described by the formal chemical composition of (SmS)_1.19_(TaS_2_) is built from NaCl-type SmS layers alternating with TaS_2_ fragments in which tantalum atoms are surrounded by slightly distorted trigonal prisms of sulfur. Both structural units can be described in the *Fm*2*m* space group with lattice parameters *a*_1_ = 5.552(3) Å, *b*_1_ = 5.679(4) Å, *c*_1_ = 22.50(4) Å for the SmS fragment, and *a*_2_ = 3.293(1) Å, *b*_2_ = 5.679(4) Å, *c*_2_ = 22.50(4) Å for TaS_2_ sub-lattice. The corresponding sub-lattice axes are parallel to each other, and the layers are stacked in the *c*-direction. The (SmS)_1.19_TaS_2_ composition of the material is governed by the ratio *a*_2_/*a*_1_ defining the number of formula units per cell^3,12^.

Due to this structural arrangement, (REX)_1 + *x*_(MX_2_) are frequently denoted as misfit materials^3,4^, and the presence of chemically and structurally dissimilar 2D fragments in their crystal lattices is responsible for the emergence of intriguing physical properties, including superconductivity^13^, metallicity, semiconductivity^3,14,15^, and diverse magnetism^3,16,17^, among others^4,12,18^. The majority of (REX)_1 + *x*_(MX_2_) known to date have been synthesized from pure elements, metals, and chalcogens, by heating them in sealed ampules in the presence of halogens or metal halides as transport agents^3,4,19–21^. As exact compositions of such complex multi-component materials are hard to control during high-temperature synthesis, the conventionally prepared heterostructures are routinely denoted by the theoretical (limiting) compositions of the respective misfit compounds. In a few instances, when (REX)_1 + *x*_(MX_2_) materials were investigated by quantitative analytical techniques, as-obtained RE:M:X ratios deviated from the expected (theoretical misfit) values, indicating compositional irregularities in both REX and MX_2_ sub-lattices. In addition, the studied compounds contained noticeable amounts of halogens that served as transport agents during their synthesis, complicating the analytical results^3,4,11,20,22^.

Below, we describe an example of the mechanochemical solid-state generation of (REX)_1 + *x*_(MX_2_) heterostructures under ambient conditions, whereby (REX)_1 + *x*_(MX_2_) form as spatially disorganized nano-architectures. Their crystallinity substantially improves upon subsequent annealing that, however, does not alter their internal atomic arrangements, resembling in a way the behavior of mechanochemically generated molecular solids^23^ or coordination polymers^24^. Density-functional theory (DFT) calculations provide valuable insight into the discovered synthetic phenomenon and reveal details about interatomic forces that guide otherwise stochastic mechanochemical processes toward self-organization. To avoid confusion while describing our materials synthesis, the 3D heterostructures discussed are denoted as (REX)_*n*_(MX_2_), where *n* reflects the molar amount of the REX compound, which is combined with one molar equivalent of MX_2_ during synthesis. For the theoretical misfit compositions, *n* = 1 + *x*, where *x* is calculated as noted above^11^.

## Results

### Mechanochemical synthesis

**Fig. 2:**
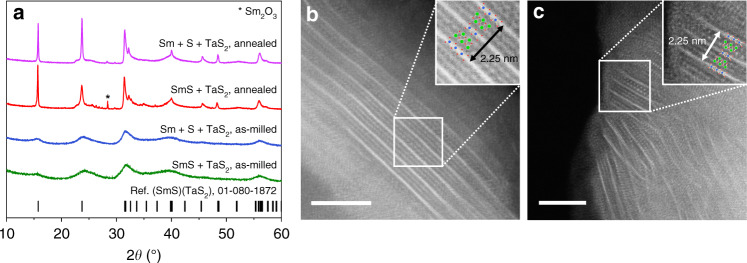
Mechanochemical synthesis of the (SmS)(TaS_2_) heterostructure. **a** PXRD patterns of (SmS)(TaS_2_) obtained by ball milling of SmS and TaS_2_, or Sm, S, and TaS_2_ in the planetary mill for 30 h, and of the respective materials annealed at 1000 °C for 3 days. PXRD patterns of the starting materials, SmS and TaS_2_, are presented in Supplementary Information. **b**, **c** HAADF-STEM images of respective as-milled samples. The insets display enhanced views of the 3D heteroatomic moieties. Scale bars, 5 nm.

Processing of an equimolar mixture of SmS and TaS_2_ in a planetary mill for 30 h produces a powder whose powder X-ray diffraction (PXRD) pattern (Fig. 2a) does not contain Bragg peaks of the starting materials indicating their complete conversion into the reaction products (Supplementary Figs. 1 and 2a). Instead, it consists of a set of broad Bragg reflections, positions of which agree with those expected for a (SmS)_*n*_(TaS_2_)-type heterostructure. High-temperature annealing improves the crystallinity of the as-milled sample and significantly reduces the width of the Bragg reflections in its PXRD pattern without shifting their positions. The prepared (SmS)(TaS_2_) is isostructural to (PbSe)_1.13_TaS_2_^25^ and its REX and MX_2_ building blocks crystallize in the *Fm*2*m* space group. The relationship between the unit-cell parameters in (SmS)(TaS_2_) is defined by the space fitting of the structurally dissimilar SmS and TaS_2_ fragments, leading to incommensurate modulation distorting periodicity along the *a* crystallographic direction (Fig. 1). The structural parameters determined for as-milled and annealed (SmS)(TaS_2_), as well as the modulation factors *α* = *a*[REX]/*a*[MX_2_], are listed in Table 1. Remarkably, they are in good agreement with the values reported in the literature as (SmS)_1.19_(TaS_2_)^19,26^.

**Table 1:** (REX)_*n*_(MX_2_) heterostructures synthesized in this work.

Material	As-milled (Å)^a^	As-milled *a*_1_/*a*_2_	Annealed (Å)^a^	Annealed *a*_1_/*a*_2_	Lit.^19,20,26^ (Å)^a^	Lit.^19,20,26^ *a*_1_/*a*_2_
(SmS)(TaS_2_)	*a*_1_ = 5.540(1)	1.689	*a*_1_ = 5.556(1)	1.685		
	*a*_2_ = 3.279(1)		*a*_2_ = 3.297(1)			
	*b* = 5.678(1)		*b* = 5.675(1)		^b^	^b^
	*c* = 22.514(3)		*c* = 22.509(3)			
(SmS)_1.19_(TaS_2_)			*a*_1_ = 5.549(1)	1.675	*a*_1_ = 5.552(3)	1.686
			*a*_2_ = 3.311(1)		*a*_2_ = 3.293(1)	
	^c^	^c^	*b* = 5.669(1)		*b* = 5.679(4)	
			*c* = 22.477(3)		*c* = 22.50(4)	
(LaSe)(NbSe_2_)			*a*_1_ = 6.025(1)	1.756		
			*a*_2_ = 3.431(1)			
	^c^	^c^	*b* = 6.075(1)		^b^	^b^
			*c* = 24.039(1)			
(LaSe)_1.14_(NbSe_2_)			*a*_1_ = 5.952(1)	1.722	*a*_1_ = 6.016(3)	1.755
			*a*_2_ = 3.457(1)		*a*_2_ = 3.428(3)	
	^c^	^c^	*b* = 6.083(1)		*b* = 6.070(3)	
			*c* = 24.075(1)		*c* = 24.072(3	
(LaSe)(TaS_2_)	*a*_1_ = 5.558(5)	1.620	*a*_1_ = 5.579(1)	1.640		
	*a*_2_ = 3.430(4)		*a*_2_ = 3.401(1)			
	*b* = 5.616(6)		*b* = 5.617(1)		^b^	^b^
	*c* = 23.337(11)		*c* = 23.422(1)			
(LaSe)_1.09_(TaS_2_)			*a*_1_ = 5.574(1)	1.629		
			*a*_2_ = 3.421(1)			
	^c^	^c^	*b* = 5.585(1)		^b^	^b^
			*c* = 23.321(1)			
LaTa(Se_0.5_S_0.5_)_3_			*a*_1_ = 5.434(3)	1.587		
			*a*_2_ = 3.423(1)			
	^c^	^c^	*b* = 5.857(3)		^b^	^b^
			*c* = 23.616(3)			

^a^Lattice parameters; standard deviations are given within parentheses.

^b^Data are unavailable.

^c^Structure is not refined due to strong peak broadening.

(SmS)(TaS_2_) also forms when TaS_2_ is combined with the equimolar amounts of pure Sm and S (1:1:1 molar ratio) and processed according to the identical preparation protocol. Both as-milled and annealed materials are practically indistinguishable from those prepared from binary compounds (Fig. 2a), suggesting a similar reaction pathway in both cases. Given the fact that SmS easily forms upon ball milling of Sm and S (Supplementary Fig. 2a), the formation of a SmS intermediate during mechanical processing of Sm, S, and TaS_2_ is a highly feasible first step. Also notable is the fact that although (SmS)(TaS_2_) has been prepared with a significant (∼19 mol.%) excess of TaS_2_, no residual precursors could be detected in this material before and after the annealing, which indicates their complete conversion into the reaction products.

**Fig. 3:**
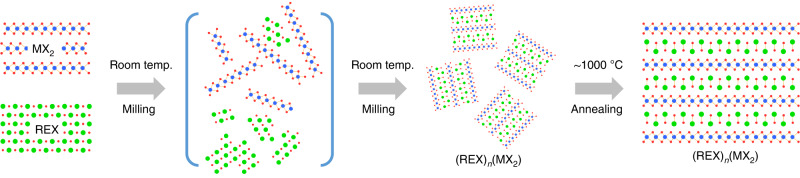
Schematic representation of 3D heterostructures synthesis. Mechanochemical synthesis of (REX)_*n*_(MX_2_) heterostructures, where RE = rare earth; M = Ta or Nb; X = S or Se; and *n* values vary between 1 and 1.2.

A sample with theoretical misfit composition (SmS)_1.19_(TaS_2_)^19,26^ was also synthesized by ball milling SmS and TaS_2_ combined in a 1.19:1 molar ratio. While as-milled and annealed solids are similar to (SmS)(TaS_2_), still unreacted exfoliated TaS_2_ nanosheets are present in the as-milled sample (Supplementary Fig. 3b), which is in line with the reaction mechanism depicted in Fig. 3. Further, the annealed (SmS)_1.19_(TaS_2_) contains a distinct secondary phase in its PXRD pattern. This minor, most likely samarium-oxysulfide impurity, is not present in the starting materials or in the (SmS)(TaS_2_) product.

The high-angle annular dark-field scanning transmission electron microscopy (HAADF-STEM) and energy-dispersive X-ray spectroscopy (EDS) experiments shed light on this mystery. As evident from Fig. 2b, c (and Supplementary Fig. 3b, c) as-milled (SmS)(TaS_2_) and (SmS)_1.19_(TaS_2_) heterostructures are composed from similar moieties. Their spatial alignments substantially improve upon annealing, which is in line with PXRD data. The interlayer TaS_2_–TaS_2_ distances within the separate heterostructured moieties remain unaffected by heating, hence suggesting similarity of the atomic arrangements in as-milled and annealed materials.

**Fig. 4:**
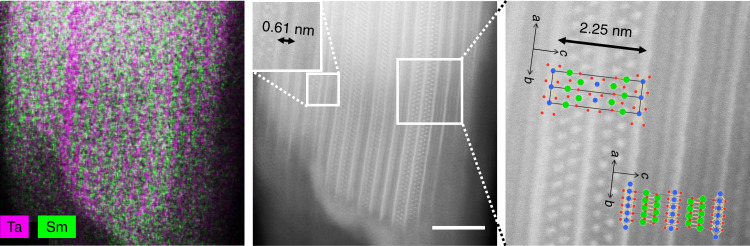
Microscopy characterization of the (SmS)(TaS_2_). STEM-EDS and HAADF-STEM images of (SmS)(TaS_2_) synthesized by ball milling and annealing of SmS and TaS_2_ mixture. A broad pink line in the EDS elemental mapping indicates a TaS_2_–TaS_2_ double layer that is clearly seen in the HAADF-STEM image (interlayer spacing 0.61 nm). Scale bar, 5 nm.

Detailed HAADF-STEM and EDS investigations (Fig. 4) of a favorably oriented particle of the annealed (SmS)(TaS_2_) reveal the presence of a TaS_2_–TaS_2_ double layer, suggesting that the excess of TaS_2_ used for its preparation is incorporated into the final product as adjacent TaS_2_–TaS_2_ layers, representing stacking faults. Similar structural arrangements have been observed in other particles of the mechanochemically prepared (SmS)(TaS_2_) (Supplementary Fig. 4), and also reported in the literature for (BiS)(VS_2_)_*n*_ misfit materials (1 ≤ *n* ≤ 15)^27^. Besides stacking faults, vacancies, antisites, and other defects may also contribute to compositional irregularities of misfit compounds.

Thus, our results unambiguously confirm the formation of 3D misfit heterostructures upon mechanochemical disassembly and re-ordering of binary metal chalcogenide precursors. The suitable nano-sized building blocks can be also generated in situ from the elements; hence, it is expected that mechanical processing of Ta, Sm, and S would produce (SmS)_*n*_(TaS_2_) hetero-assemblies as well.

To test this hypothesis, we ball milled a mixture of Sm, Ta, and S, combined in the 1:1:3 molar atomic ratio, in a shaker mill for 12 h. Then, the as-milled powder was studied using HAADF-STEM and EDS elemental mapping (Supplementary Fig. 5), which confirms the presence of 3D heterostructures in the sample. However, Ta is incompletely dispersed in the as-milled material and Ta-rich spots remain visible in its EDS mapping (Supplementary Fig. 5c). The high-temperature annealing produces (SmS)(TaS_2_) identical to that made from other precursors.

The results are quite remarkable as ball milling of elemental Ta and S does not produce detectable amounts of TaS_2_ even after prolonged processing (Supplementary Fig. 6). Therefore, it may be anticipated that SmS, mechanochemically generated at the early stages of the reaction, serves as a “scavenger” for {TaS_*x*_} species, which form on the surface of the metallic Ta that is exposed to sulfur during mechanical processing. Alternatively, SmS may serve as a template for converting {TaS_*x*_} into TaS_2_ layers of the thermodynamically stable (SmS)_*n*_(TaS_2_) heterostructure (Supplementary Fig. 7).

**Fig. 5:**
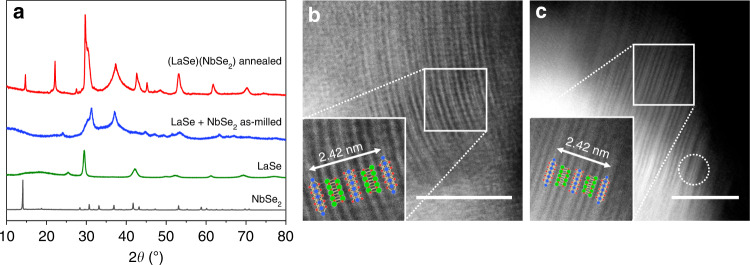
Synthesis of the (LaSe)(NbSe_2_) heterostructure. **a** PXRD patterns and **b** HAADF-STEM images of (LaSe)(NbSe_2_) obtained by ball milling LaSe and NbSe_2_ for 12 h; **c** the HAADF-STEM image of the annealed material. An NbSe_2_–NbSe_2_ double layer is recognized in the lower right-hand corner of the image. Insets: enhanced atomic-scale views of 3D heterostructural arrangements. Scale bars, 5 nm.

Very similar mechanochemical transformations are observed when an equimolar (1:1 molar ratio) mixture of LaSe and NbSe_2_ is ball milled in a shaker mill for up to 12 h. In this case, the as-milled powder consists of disordered (LaSe)(NbSe_2_) fragments, which further align upon annealing (Fig. 5b, c). Further structural analysis of the annealed (LaSe)(NbSe_2_) reveals that it is isostructural to (PbSe)_1.10_NbSe_2_^25^ and its lattice parameters (Table 1) agree well with those previously reported for (LaSe)_1.14_(NbSe_2_)^20^.

An attempt to mechanochemically prepare (LaSe)_1.14_(NbSe_2_) by combining LaSe and NbSe_2_, taken in the 1.14:1 molar ratio, produced a powder material that further crystalizes upon heating. Similar to (SmS)_1.19_(TaS_2_), the annealed (LaSe)_1.14_(NbSe_2_) also contains a minor secondary phase that does not belong to the precursor materials (Supplementary Fig. 8).

To explore further the scope of the mechanochemically facilitated 3D heterostructuring, we applied the newly developed synthetic approach to the synthesis of (LaSe)_*n*_(TaS_2_), where sulfur and selenium are expected to occupy different sites in adjacent TaS_2_ and LaSe sub-lattices. The PXRD patterns of both as-milled and annealed (LaSe)(TaS_2_) samples prepared from equimolar amounts of LaSe and TaS_2_ indicate the formation of 3D heterostructures (Supplementary Fig. 9) with lattice parameters falling between those of (LaSe)(NbSe_2_) and (SmS)(TaS_2_) (Table 1). Even after 10 days of holding at 1000 °C, the positions of Bragg peaks of the annealed (LaSe)(TaS_2_) material remain unchanged (Supplementary Fig. 10).

Subsequently, the sample with the theoretical misfit stoichiometry, (LaSe)_1.09_(TaS_2_), was also prepared from LaSe and TaS_2_ combined in the 1.09:1 molar ratio. Also in this case, the lattice parameters of the annealed (LaSe)_1.09_(TaS_2_) are very close to those of (LaSe)(TaS_2_) (Table 1) and a minor secondary phase impurity is found in the material (Supplementary Fig. 11a–c).

Unfortunately, STEM-EDS, Raman, and solid-state nuclear magnetic resonance (SSNMR) spectroscopies were unable to confirm unambiguously the exact locations of S and Se in the crystal lattice of (LaSe)_*n*_(TaS_2_). Therefore, we synthesized a LaTa(Se_0.5_S_0.5_)_3_ misfit material with a statistical distribution of S and Se by reacting elemental La, S, Se, and Ta, taken in 1:1:1.5:1.5 atomic ratio, using processing conditions identical to those developed during the preparation of (SmS)(TaS_2_) from elements (Table 1 and Supplementary Fig. 11d–f).

The lattice parameters of the annealed LaTa(Se_0.5_S_0.5_)_3_, especially the *a* parameter corresponding to the LaX sub-lattice, considerably deviate from those of (LaSe)(TaS_2_), suggesting different chalcogen occupancies in LaTa(Se_0.5_S_0.5_)_3_ and (LaSe)_*n*_(TaS_2_) heterostructures, which may be explained by assuming the preferential location of Se in the LaX sub-lattice of (LaSe)_*n*_(TaS_2_). Remarkably, mixing of La, Ta, S, and Se in the 1:1:1.5:1.5 proportion and heating them without milling at 1000 °C for 3 days does not produce LaTa(Se_0.5_S_0.5_)_3_ (Supplementary Fig. 12), thus confirming the critical role of milling in the synthetic process.

### ^77^Se solid-state NMR and Raman spectroscopic studies

Structural models derived from PXRD and STEM experiments were further explored using ^77^Se SSNMR and Raman spectroscopy. The ^77^Se SSNMR spectra of the Se-containing (REX)_*n*_(MX_2_) heterostructures and binary metal chalcogenides LaSe, NbSe_2_, and TaSe_2_ are shown in Supplementary Fig. 13. The relatively narrow signal in the spectrum of LaSe is consistent with its highly symmetric cubic structure, where each Se is surrounded by six La atoms and the isotropic ^77^Se chemical shift of ca. 500 p.p.m. is within the range of chemical shifts previously reported for metal selenides^28^. The ^77^Se SSNMR spectra of both NbSe_2_ and TaSe_2_ exhibit broad axially symmetric powder patterns and their isotropic chemical shifts are in a much more positive range than that of LaSe (Supplementary Fig. 13a) or other semiconducting and insulating metal selenides^28^. The ^77^Se SSNMR spectrum of commercial NbSe_2_ matches that previously reported^29^. Borsa et al.^30^ attributed the large positive ^77^Se isotropic shift of NbSe_2_ to a Knight shift, while Smith and co-workers^29^ attributed the positive ^77^Se isotropic shift to the hyperfine coupling between Se and unpaired *d* electrons residing on Nb(IV) atom^29^. The significant hyperfine shift anisotropy/Knight shift anisotropy/chemical shift anisotropy of ca. 2000 p.p.m. observed for both NbSe_2_ and TaSe_2_ is also consistent with the trigonal pyramidal coordination of selenium atoms in these layered TMDCs.

The ^77^Se SSNMR spectra (Supplementary Fig. 13b) of (LaSe)_*n*_(NbSe_2_), (LaSe)_*n*_(TaS_2_), and LaTa(S_0.5_Se_0.5_)_3_ show no signals corresponding to the binary TMDCs, thus confirming complete conversion of the starting materials to products. The disappearance of the large positive shifts from the ^77^Se SSNMR spectra of the misfit heterostructures can be attributed to the electron transfer between their REX and MX_2_ sub-lattices, which is also in line with the results of the DFT calculations presented below. The ^77^Se SSNMR spectra of the (LaSe)_*n*_(NbSe_2_) materials show anisotropic shielding patterns with breadths similar to those observed for the pure starting compounds. Se atoms in these heterostructures are situated in both Nb and La sub-lattices, and, as the symmetry of Se atoms in the NbSe_2_ layers of the misfit material remains largely unchanged, the magnitude of the shielding anisotropy is expected to be similar to that observed in the pure TMDC. While the ^77^Se SSNMR spectrum of LaSe produces a relatively narrow isotropic signal, the symmetry of Se atoms is reduced (Fig. 1) in the misfit heterostructures, leading to an anisotropic signal broadening. Consequently, it is challenging to assign definitively the ^77^Se SSNMR signals and to quantify the Se occupancy in the different sub-lattices by NMR spectroscopy (Supplementary Fig. 13).

Raman spectroscopy also confirms the absence of starting materials in all as-milled and annealed samples. Five characteristic bands are observed in the spectrum of (SmS)(TaS_2_) (Supplementary Fig. 14a) with the bands at 119 and 148 cm^−1^ corresponding to intralayer vibrations of the SmS sub-lattice. The higher energy bands at 302 and 327 cm^−1^ can be assigned to the intralayer *E* vibrational modes of the hexagonal TaS_2_ sub-lattice that are upshifted compared to the bulk TaS_2_. The band at 397 cm^−1^ is probably associated with intralayer *A* vibrations in TaS_2_, which, in contrast to the *E* mode, appear at the same position as *A*_1*g*_ vibrations in the pristine TaS_2_^31,32^. Similar peaks, shifted toward the lower wavenumbers, are also observed in the spectra of (LaSe)(NbSe_2_) and (LaSe)(TaS_2_) (Supplementary Fig. 14b, c). In all studied cases, the Raman spectra of the materials with the compositions (REX)_*n*_(MX_2_) (Supplementary Fig. 14d–f) are very similar to those of the respective (REX)(MX_2_) heterostructures, which is in line with the results of our PXRD and STEM data.

### DFT calculations

DFT calculations were used to assess stabilities of (REX)_*n*_(MX_2_) heterostructures with limiting nominal compositions (*n* = 1.14 for RE = La, M = Nb, X = Se, and *n* = 1.19 for RE = Sm, M = Ta, and X = S). Calculation details are included into Supplementary Note 1. The total energy (*E*_tot_) and formation energy (*E*_form_) for each phase are listed in Table 2, as well as relative energies Δ*E* to highlight the relative stability of the heterostructures. Recall: *E*_form_ is the total energy relative to the weighted probabilities of the constituent-elemental energies; it is the energy gained [or lost] in the formation of the heterostructure, as measured in calorimetry, for example, *E*_tot_ is the energy of the heterostructure relative to atomic vacuum.

**Table 2:** Total (*E*_total_) and formation (*E*_form_) energies of (REX)_*n*_(MX_2_) heterostructures with comparison to cubic-REX and 2H-MX_2_ chalcogenides^a^.

System	*E*_total_ (eV/atom)	Δ*E* (eV/atom)	*E*_form_ (eV/atom)	System	*E*_total_ (eV/atom)	Δ*E* (eV/atom)	*E*_form_ (eV/atom)
Cubic-SmS	−6.4487	+0.759	−2.0248	Cubic-LaSe	−6.2077	+0.385	−1.9948
2H-TaS_2_	−7.2078	0	−0.5027	2H-NbSe_2_	−6.5929	0	−0.8542
(SmS)_1.19_(TaS_2_)	−7.3708	−0.163	−1.5781	(LaSe)_1.14_(NbSe_2_)	−6.6141	−0.021	−1.48570

^a^The energy Δ*E* is referenced to 2H-MX2 to assess the relative stability for the heterostructures, as shown in Fig. 6.

**Fig. 6:**
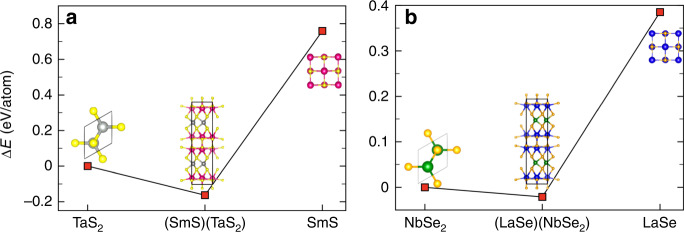
DFT calculations for (REX)_*n*_(MX_2_) heterostructures. **a** Relative DFT energies Δ*E* (i.e., the difference of total energies per atom relative to 2H-MX_2_) of (SmS)_*n*_(TaS_2_), *n* = 1.19 and of **b** (LaSe)_*n*_(NbSe_2_), *n* = 1.14 misfit compounds compared to single-phase cubic-REX [SmS, LaSe] and 2H-MX_2_ [TaS_2_, NbSe_2_] building blocks.

The relative energy Δ*E* (per atom) for cubic-(REX) and (REX)_*n*_(MX_2_) with respect to 2H-MX_2_ is plotted in Fig. 6. The relative energies (total energies *E*_tot_) of (SmS)_1.19_(TaS_2_) and (LaSe)_1.14_(NbSe_2_) are −0.163 eV/atom (−1.578 eV/atom) and −0.021 eV/atom (−1.486 eV/atom), respectively, see Table 2. Δ*E*s are significantly lower than their binary precursors, that is, at −0.922 eV/atom below SmS and −0.163 eV/atom below TaS_2_ for (SmS)_1.19_(TaS_2_), and, similarly, at −0.406 eV/atom below LaSe and −0.021 eV/atom below NbSe_2_ for (LaSe)_1.14_(NbSe_2_). This relative energy shows that mixing of MX_2_ and REX lowers the total energy of the misfit compounds, and the negative *E*_form_ of (SmS)_1.19_(TaS_2_) and (LaSe)_1.14_(NbSe_2_) in Table 2 further shows the increased stability of misfit compounds. (REX)_*n*_(MX_2_) heterostructures are layered MX_2_ materials intercalated by REX compounds, where metal atoms (M) adopt a +4-oxidation state, RE elements assume a +2-oxidation state, and chalcogens (X) have −2-oxidation state. As it intercalates, their structure can be stabilized by electron transfer between the REX fragment and the MX_2_ network^33^. (SmS)_1.19_(TaS_2_) and (LaSe)_1.14_(NbSe_2_) represent good examples of such incommensurate misfit materials, and their electronic structures will be discussed as two representative cases of S- and Se-containing heterostructures.

**Fig. 7:**
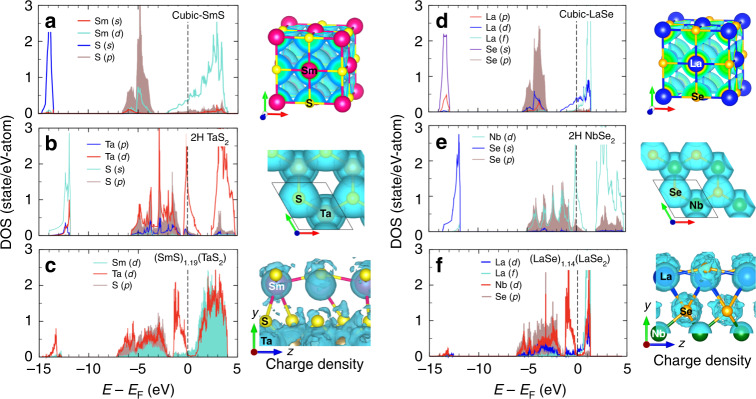
DOS calculations for (REX)_*n*_(MX_2_) heterostructures. **a** Partial DOS and charge density of cubic-SmS, **b** 2H-TaS_2_, **c** (SmS)_1.19_(TaS_2_), **d** cubic-LaSe, **e** 2H-NbSe_2_, and **f** (LaSe)_1.14_(NbSe_2_). Charge density isosurfaces (0.03 *e*^−^/Å^3^) are plotted to highlight the distribution.

The density of states (DOS) of binary precursors, SmS and TaS_2_, is shown in Fig. 7a, b. The sulfur *s*-bands in SmS are far below the Fermi energy (*E*_F_), separated from the other valence states by 7.0 eV. The bands near *E*_F_ mainly consist of sulfur *p-*bands that are weakly hybridized with Sm *d-*orbitals. The weak or lack of hybridization between Sm *s-* and *d-*bands and S *p-*orbitals is evident from non-overlapping (spherical) charge density of Sm and S atoms, suggesting dominance of ionic bonding in SmS.

In contrast, the DOS of 2H-TaS_2_ (Fig. 7b) shows two separate sets of energy bands near E_F_ with the energy width of ∼ 1 eV, which are formed by the crystal field created by octahedrally (prismatic) arranged S around Ta. The DOS associated with Ta *d*-band shows a strong maximum near *E*_F_ and the bands just below *E*_F_ consist of the S *p-*orbitals with a non-negligible contribution from Ta *d*-orbitals, whereas the lowest-energy bands consist of S *s-*orbitals and are located at −12.5 eV.

In (SmS)_1.19_(TaS_2_), the S *p-*bands and Sm *d-*bands are shifted compared to those in the parent 2H-TaS_2_ and cubic-SmS materials (Fig. 7c). A fraction of their shift can be attributed to higher filling of $d_{z^2}$-state in Ta, while other components of the shift occur by lowering the energy of S *s*-orbitals due to Coulombic interactions with Sm. The sulfur *s-*band contributed by the SmS fragment is shifted toward lower energies (near −16.5 eV below *E*_F_) with respect to the parent cubic-SmS, most likely due to the presence of an electron in the Sm *d-*band. The set of bands between *E*_F_ and −1.5 eV corresponds to $d_{z^2}$*-*states in Ta, whose bandwidth is similar to that in the parent TaS_2_. The lower edge of the $d_{z^2}$-band in (SmS)_1.19_(TaS_2_) is at 0.5 eV below *E*_F_, while it crosses *E*_F_ in 2H-TaS_2_. Thus, the $d_{z^2}$*-*band in (SmS)_1.19_(TaS_2_) is partially occupied with electron density transferred from SmS to the TaS_2_ sub-lattice. In binary TaS_2_, the Ta $d_{z^2}$*-*band is half filled (i.e., one hole per Ta atom). However, a Bader charge analysis^34^ of (SmS)_1.19_(TaS_2_) (Supplementary Table 3 and Supplementary Figs. 15 and 16) reveals that, in fact, the Ta $d_{z^2}$-band contains only 0.17–0.21 holes per Ta, indicating a charge transfer of 0.83–0.79 from cubic-SmS to 2H-TaS_2_. The charge density isocontour (0.03 *e*^−^/Å^3^) is shown in Fig. 7c and highlights the interlayer coupling in (SmS)_1.19_(TaS_2_) mediated by this charge transfer. The charge densities are shown between −2 eV to *E*_F_. They indicate a significant charge density overlap along Sm-S-Ta bonding directions via the S atom that belongs to the TaS_2_ layer. The Bader analysis further confirms that charge transfer can stabilize the incommensurate (REX)_*n*_(MX_2_) systems (Supplementary Table 3 and Supplementary Figs. 15 and 16).

The electronic structures of cubic-LaSe, 2H-NbSe_2_, and (LaSe)_1.14_(NbSe_2_) are similar to (SmS)_1.19_(TaS_2_) and its building blocks, see Fig. 7d–f. The energy gap between Se *s-* and *p-*bands in (LaSe)_1.14_(NbSe_2_) is ~3.4 eV, smaller than the corresponding values for cubic-LaSe (7.5 eV) and 2H-NbSe_2_ (6.80 eV). In (LaSe)_1.14_(NbSe_2_), two bands at −6.5 and −1.60 eV are formed from Se *p-*bands and *d-*bands of La and Nb, while the bands between −1.4 and 1.3 eV originate from Nb $d_{z^2}$*-*orbital that, in turn, is hybridized with Se *p*-states and La *d-*states. The Nb $d_{z^2}$*-*band contains ∼0.2 holes per Nb atom, indicating a transfer of ∼0.8 electrons from LaSe to NbSe_2_. In this case, charge transfer proceeds through Se atom and serves as a “glue” for the entire hetero-assembly.

In summary, DFT calculations confirm that charge transfer from REX to MX_2_ stabilizes (REX)_*n*_(MX_2_) misfit compounds, and glues together the structurally different elements of such heterostructures. The directionality of the interlayer charge densities suggests that the bonding character is largely covalent in misfit compounds and the effective charge sharing increases the bonding strength, which lowers the free energy of misfit materials with respect to binary REX and MX_2_ phases. This helps to understand the formation of the (REX)_*n*_(MX_2_) heterostructures from both binary metal chalcogenides and the elements, for (REX)_*n*_(MX_2_) is the most thermodynamically stable atomic arrangement in the studied RE-M-3X systems. Finally, our calculations identify the chalcogen atoms as conduit for the charge transfer in (REX)_*n*_(MX_2_)—a remarkable result not reported before, to the best of our knowledge.

### Electronic transport studies

Investigations of the electronic transport in (REX)_*n*_(MX_2_) prepared in this work provide further valuable information about their physical and chemical nature and serve as an additional marker for consistency of the developed synthetic approach.

**Fig. 8:**
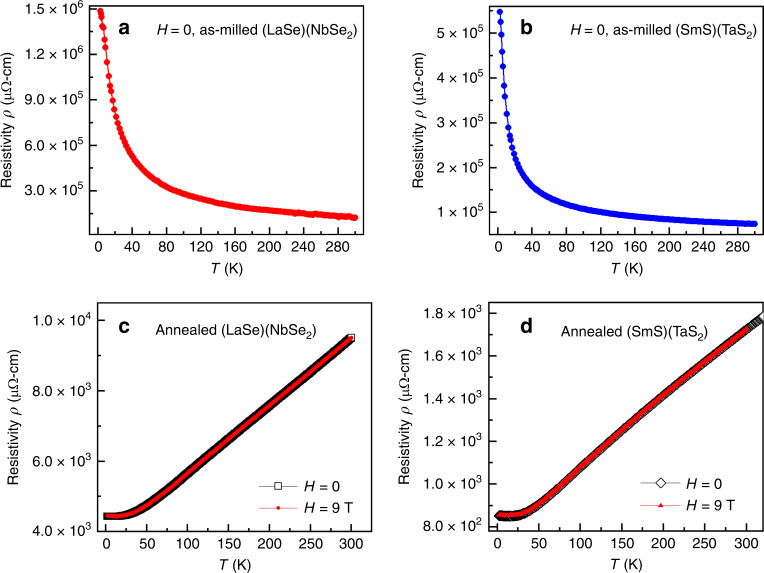
Electrical resistivity characterization of heterostructures. **a** Electrical resistivity versus temperature measured in 0 and 9 T fields for as-milled and **c** annealed (LaSe)(NbSe_2_); **b** as-milled and **d** annealed (SmS)(TaS_2_).

The temperature dependencies of the electrical resistivities, *ρ*(T), for as-milled (LaSe)(NbSe_2_) and (SmS)(TaS_2_), measured between 300 and 2 K in the absence of external magnetic field (*H* = 0), are shown in Fig. 8. The resistivity values observed are between 0.12 Ω-cm (LaSe)(NbSe_2_) and 0.074 Ω-cm (SmS)(TaS_2_) at 300 K. They slowly increase with decreasing temperature and reach 0.45 Ω-cm for (LaSe)(NbSe_2_) at 50 K and 0.18 Ω-cm for (SmS)(TaS_2_) at 30 K.

Around 30–50 K, the resistivities of both as-milled materials begin to grow exponentially, indicating possible transition from semiconducting to insulating states that has not been reported for misfit heterostructures in the past and is not observed in the annealed samples synthesized in this work. The nature of the discovered phenomenon is quite intriguing and requires additional studies, which will be published elsewhere.

The annealed (LaSe)(NbSe_2_) and (SmS)(TaS_2_) demonstrate metallic behavior with electrical resistivities of 9.5 mΩ-cm for (LaSe)(NbSe_2_) and 1.7 mΩ-cm for (SmS)(TaS_2_) at 300 K, that is, ∼ 10 and 40 times lower values than those observed for as-milled samples. They further decrease with decreasing temperature and reach 4.4 mΩ-cm for (LaSe)(NbSe_2_) at 12 K and 0.8 mΩ-cm for (SmS)(TaS_2_) at 15 K (Fig. 8c, d). The annealed (LaSe)_1.14_(NbSe_2_) demonstrates transport behaviors similar to those of the annealed (LaSe)(NbSe_2_) (Supplementary Fig. 17). The transport properties of the annealed (SmS)(TaS_2_) material are in line with that previously reported for a single-crystal of (SmS)_1.19_(TaS_2_)^21^. Strong external magnetic field of 9 T does not show a measurable effect on the electronic transport of the investigated samples.

Both annealed (LaSe)_*n*_(TaS_2_) materials also display metallic behaviors that do not depend on their formal chemical composition (Supplementary Fig. 18) and resemble that of (SmS)(TaS_2_) and (LaSe)(NbSe_2_). However, unlike the latter, (LaSe)_*n*_(TaS_2_) exhibit distinct resistivity drops ~4 K that coincide with superconducting transitions in metallic Ta^35,36^ and TaS_1 − *x*_Se_*x*_ with low Se content, *x* < 0.2^37^—trace impurities that may have formed during the heat treatment. Thus, electronic transport of all studied annealed materials shows remarkably little dependence on their formal chemical compositions and is in line with data reported for (REX)_*n*_(MX_2_) with *n* > 1.

## Discussion

In conclusion, we demonstrate that mechanical milling, in contrast to common belief about its destructive nature, consistently and reproducibly facilitates 3D heterostructural formation via disassembly and re-ordering of structurally dissimilar RE and transition-metal chalcogenides at ambient temperature and pressure. The created misfit moieties can further structurally align under heat treatment in a way that is common for molecular solids and polymers, that is, without altering their intrinsic atomic arrangements. Distinct from conventional heterostructuring techniques, the discovered synthetic approach may enable the 3D heterostructures with site-separated components that are inaccessible via conventional synthetic routes.

Our DFT calculations uncovered unique details about chemical bonding and charge transfer in the incommensurate misfit metal chalcogenides that allows understanding the mechanochemical formation from binary metal chalcogenides or pure elements.

Detailed investigation of the chemical structures and properties of the mechanochemically synthesized misfit heterostructures uncovered a clear contradiction between anticipated stoichiometries of the (REX)_*n*_(MX_2_) solids and their experimentally achieved chemical compositions, which can be extended onto other misfit materials reported in the literature.

## Methods

### Materials

Ultra-high-purity Ar (Matheson, 99.999%), ultra-high-purity He (Matheson, 99.999%), NbSe_2_ (Alfa Aesar, 99.8% purity), La (Materials Preparation Center, Ames Laboratory [MPC]), 99.99% REE purity), Sm (MPC, REE 99.99% purity), S (Alfa Aesar, 99.9995% purity), and Se (Alfa Aesar, 99.999% purity). TaS_2_, SmS, and LaSe were prepared in-house from the elements.

### Synthesis

In a typical experiment (Fig. 3), a 2 g sample of the physical mixture of a TMDC and a RE chalcogenide (or a RE metal and a chalcogen), taken in stoichiometric proportions, was milled either in a silicon nitride milling container together with three 12.7 mm silicon nitride balls using a shaker mill (SPEX 8000M), or in a zirconia vial with five 15 mm zirconia balls using a two-station horizontal planetary mill (Fritsch, Pulverisette 7) for periods of time between 12 and 30 h. Materials handling, including loading of the vial, was performed under ultra-high-purity argon in a glove box. After ball milling, the obtained material was pressed into a pellet under argon in a glove box, placed in a quartz tube, sealed under 0.75 bar of ultra-high-purity helium, and heat treated by ramping the temperature to 1000 °C and annealing the material for 3 days. Thereafter, the sample was cooled down with the furnace to room temperature. For characterization, the prepared solid was crushed in an agate mortar with an agate pestle and stored in a glove box, both operations were performed under high-purity argon.

TMDC and RE chalcogenide precursors: NbSe_2_ was used as received. TaS_2_ was prepared by ball milling of a nearly stoichiometric mixture of tantalum and sulfur (5% excess) for 4 h in a shaker mill in a silicon nitride container followed by a 3-day heat treatment in a quartz tube sealed under 0.75 bar pressure of the ultra-high-purity helium. The PXRD analysis of the obtained material confirmed the formation of the pure TaS_2_ phase (Supplementary Fig. 1).

LaSe and SmS were prepared by ball milling of stoichiometric mixtures of RE metals and chalcogens for 12 h in a shaker mill in a silicon nitride container. The PXRD pattern contained only peaks corresponding to RE chalcogenides (Supplementary Fig. 2).

### Powder X-ray diffraction

Phase analysis and structural characterization of the reaction products were carried out using PXRD at room temperature on a PANalytical X’Pert PRO diffractometer with Cu-K*α* radiation in the range of Bragg angles 5° ≤ 2*θ* ≤ 80°. Lattice parameters were determined using Le Bail profile fitting (Supplementary Fig. 19) in FullProf^38^ with background approximated through linear interpolation between data points selected from regions free of Bragg reflections, and peak shapes approximated using pseudo-Voigt function. In the Le Bail refinements, RE chalcogenide REX (space group *Fm*2*m*) and transition-metal chalcogenide MX_2_ (space group *Cm*2*m*) sub-lattices were fitted as separate phases, each contributing its own set of Bragg reflections to the observed X-ray powder diffraction pattern. Since both commensurate axes (*b* and *c*) are identical for the two sub-lattices, their values were constrained as such during the refinements. The misfit in the structure occurs along the *a* direction; therefore, lattice parameters *a*_1_ (for REX) and *a*_2_ (for MX_2_) were refined independently for each of the sub-lattices.

### Microscopy

TEM experiments were carried out on a Titan Themis (FEI) probe Cs-corrected TEM. HAADF-STEM images were acquired with a convergence semi-angle of 18 mrad and a collection semi-angle of 99–200 mrad at 200 kV. EDS analysis was performed using a Super-X EDS detector.

### Raman spectroscopy

Powdered samples were spread on glass coverslips and analyzed with a Horiba XploRA Raman microscope (HORIBA Scientific, Edison, NJ) using 532-nm excitation (8.3 × 10^3^ W/cm^2^) and a 100× (0.90 NA) objective. The detector was a front-illuminated Horiba Synapse EMCCD camera, and the acquisition time was 60 s. For each sample, the displayed spectrum was an average of 10 locations.

### ^77^Se solid-state NMR

^77^Se SSNMR experiments were performed on a 9.4 T wide bore magnet (*v*_0_(^77^Se) = 76.34 MHz) equipped with a Bruker Avance III HD spectrometer. All spectra were obtained on an H-X double resonance Bruker static probe with a 4-mm transverse coil. Samples were packed into 4-mm zirconia rotors. Static ^77^Se SSNMR spectra were obtained with the wideband, uniform rate, smooth truncation-Carr–Purcell Meiboom–Gill (WURST-CPMG) pulse sequence^39–41^. The WURST pulses were used for excitation and refocusing. The WURST pulses were 30 μs in duration and swept over a total frequency range of 600 kHz (±300 kHz frequency offsets). This sweep width was sufficient to excite and refocus signals from paramagnetically shifted sites and cover the wide chemical shift range of ^77^Se. Spectra were obtained by processing the first full echo in the CPMG echo train. Transformation of all echoes in the CPMG echo train typically resulted in significant intensity distortions. The duration of the first spin echo was 4.5 ms. Spectra were obtained with recycle delays of 0.5 or 1 s, except for the (LaSe)_1.14_(NbSe_2_) as-milled sample for which a 60 s recycle delay was used. Between 4092 and 81,920 scans were acquired for each sample.

### DFT calculations

Electronic structure of (REX)_*n*_(MX_2_) heterostructures are calculated using the Vienna Ab-Initio Simulation Package^42,43^. The valence interaction is described by projector augmented-wave method^44^ with energy cutoff of 350 eV for the plane-wave orbitals. The Monkhorst–Pack *k*-mesh of 1 × 1 × 2 and 3 × 3 × 9 is used for Brillouin zone integration during full relaxation (lattice + ionic) and charge self-consistency (including electronic DOS)^45^, respectively; Monkhorst–Pack *k*-mesh of 25 × 25 × 25 and 19 × 19 × 11 are used for REX and MX_2_, respectively. Total energies and forces are converged to 10^−5^ eV/cell and 0.01 eV/Å. The Perdew–Burke–Ernzerhof^46^ exchange-correlation functional is employed in the generalized gradient approximation. We also perform the Bader charge analysis^34^ to determine the local charge distributions after charge self-consistency. The parent structures REX (SmS; LaSe) and MX_2_ (NbSe_2_; TaS_2_) are adopted from the Materials Project^47^. We project (REX)_*n*_(MX_2_) unit cell along (0 0 1) in the large supercell (see Supplementary Tables 1 and 2) to make the incommensurate crystal structure commensurate, and to simplify the calculation of misfit compounds.

### Electrical resistivity measurements

The electrical resistivities (*ρ*) have been measured in the absence of an external magnetic field and in the field of 9 T using a standard four-probe technique in the Physical Property Measurements System (Quantum Design, Inc.). The powdered as-milled samples are pressed with an 8-ton hydraulic press, polished and cut into rectangular pieces with 4 × 2 × 1 mm^3^ dimensions. The annealed materials are mechanically robust and could be cut to the required dimensions without additional pressing.

## Supplementary information

Supplementary Information

Peer Review File

## Data Availability

Data that support plots within this paper and other findings of this study are available from the corresponding author upon reasonable request.
