# Supplementary material for: Unprecedented generation of 3D heterostructures by mechanochemical disassembly and re-ordering of incommensurate metal chalcogenides
Source: Nat Commun. 2020 Jun 12;11:3005. doi: 10.1038/s41467-020-16672-0 (PMC7293273; doi:10.1038/s41467-020-16672-0)
Supplement: Supplementary file 1 — Supplementary Information [file 41467_2020_16672_MOESM1_ESM.pdf]

Unprecedented generation of 3D-heterostructures by mechanochemical disassembly and re-ordering of incommensurate metal chalcogenides.

Dolotko et al.

Unprecedented generation of 3D-heterostructures by mechanochemical disassembly and re-ordering of incommensurate metal chalcogenides

Oleksandr Dolotko,[1,6] Ihor Z. Hlova,[1,6] Arjun K. Pathak,[1,2,6] Yaroslav Mudryk,[1] Vitalij K. Pecharsky,[1,3] Prashant Singh,[1,6] Duane D. Johnson,[1,3] Brett W. Boote,[1,4] Jingzhe Li,[1,4] Emily A. Smith,[1,4] Scott L. Carnahan,[4] Aaron J. Rossini,[4] Lin Zhou,[1] Ely M. Eastman,[1,5] Viktor P. Balema[1]*

[1]Ames Laboratory of U.S. Department of Energy, Iowa State University, Ames, IA, 50011-2416
[2]Department of Physics, SUNY Buffalo State, Buffalo, NY, 14222
[3]Department of Materials Science and Engineering, Iowa State University, Ames, IA, 50011-1096
[4]Department of Chemistry, Iowa State University, Ames, IA, 50011-1021
[5]Reed College, Portland, OR, 97202-8199
[6]These authors contributed equally: Oleksandr Dolotko, Ihor Z. Hlova, Arjun K. Pathak, Prashant Singh
*email: vbalema@ameslab.gov

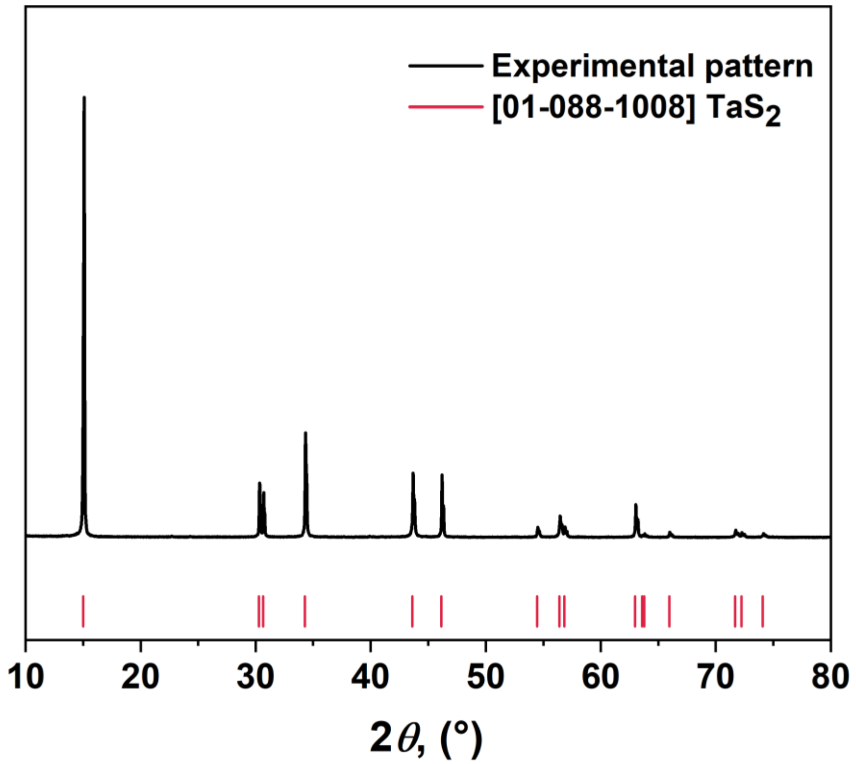

Supplementary Figure 1. PXRD pattern of TaS_2 precursor. PXRD pattern of TaS_2 prepared from the elements. Vertical bars at the bottom of the chart correspond to Bragg peak positions of TaS_2 (reference pattern PDF #01-088-1008, ICDD, 2019).

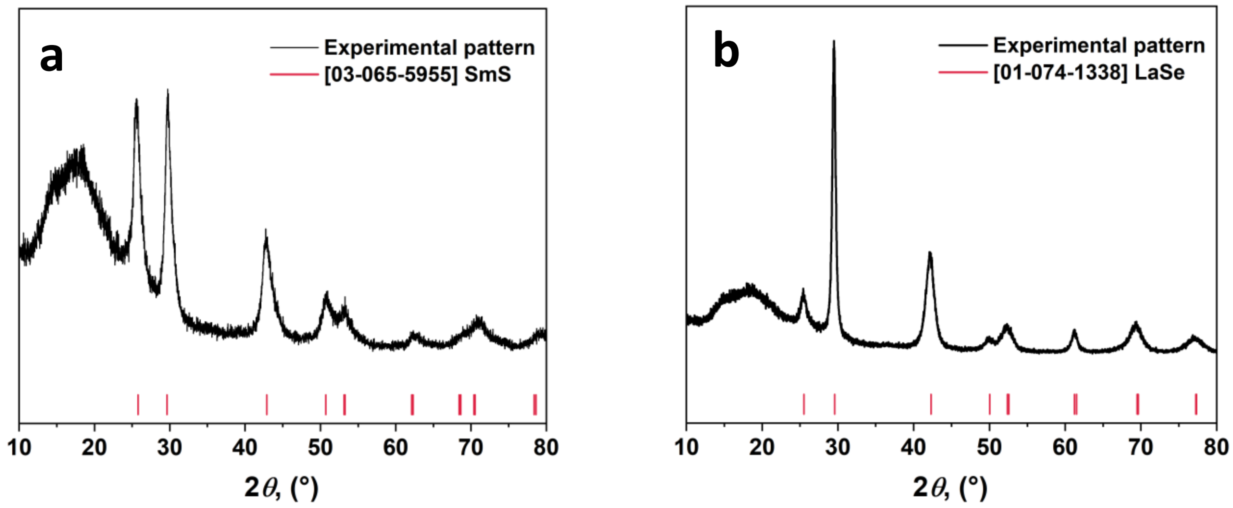

Supplementary Figure 2. **PXRD patterns of SmS and LaSe precursors. a** PXRD pattern of SmS and **b** LaSe prepared from the elements. Vertical bars at the bottom of the trace denote Bragg peak positions of corresponding chalcogenides (reference patterns PDF #03-065-5955 and PDF #01-074-1338, ICDD, 2019). Kapton film, used to protect the samples from air, adds amorphous-like background in the PXRD patterns, most prominently in the 13° ≤ 2θ ≤ 21° region.

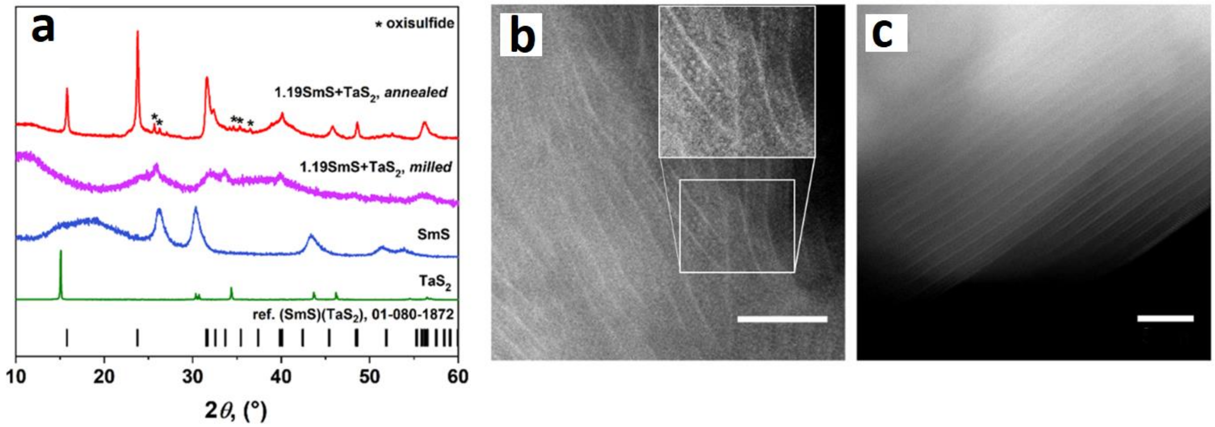

Supplementary Figure 3. Characterization of the $(SmS)_{1.19}(TaS_2)$ heterostructure. a PXRD patterns of $(SmS)_{1.19}(TaS_2)$ created by ball-milling of SmS and TaS_2 in the planetary mill for 30 hours and after annealing at 1000°C for 3 days; **b** HAADF-STEM images of the samples obtained after ball-milling and **c** annealing are shown in (b) and (c), respectively. The inset in the image (b) shows intermediately exfoliated TaS_2 nanosheets. Scale bars, 5nm.

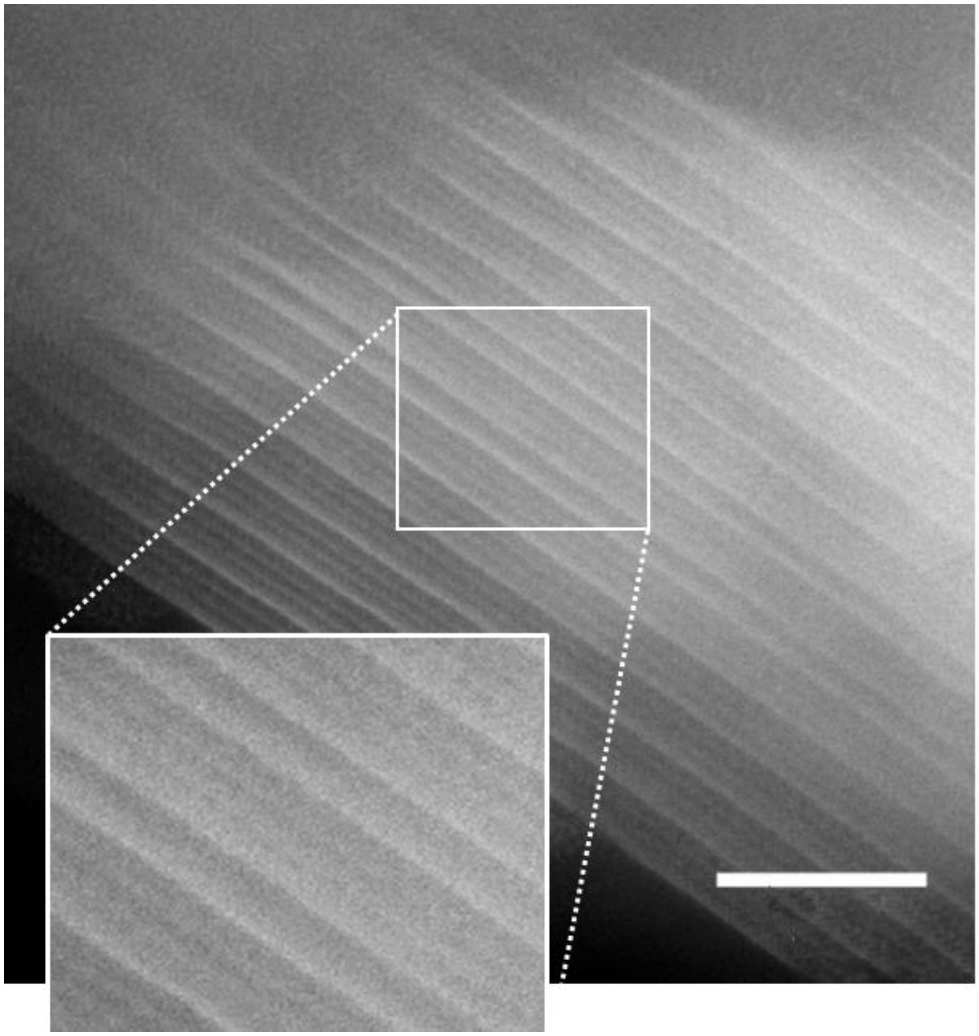

Supplementary Figure 4. Microscopy characterization of the $(SmS)_{1.19}(TaS_2)$ heterostructure. HAADF-STEM image of $(SmS)_{1.19}(TaS_2)$ synthesized by ball-milling and subsequent annealing of Sm, S and TaS_2. The expanded region visualizes the presence of TaS_2–TaS_2 double layers. Scale bar, 5nm.

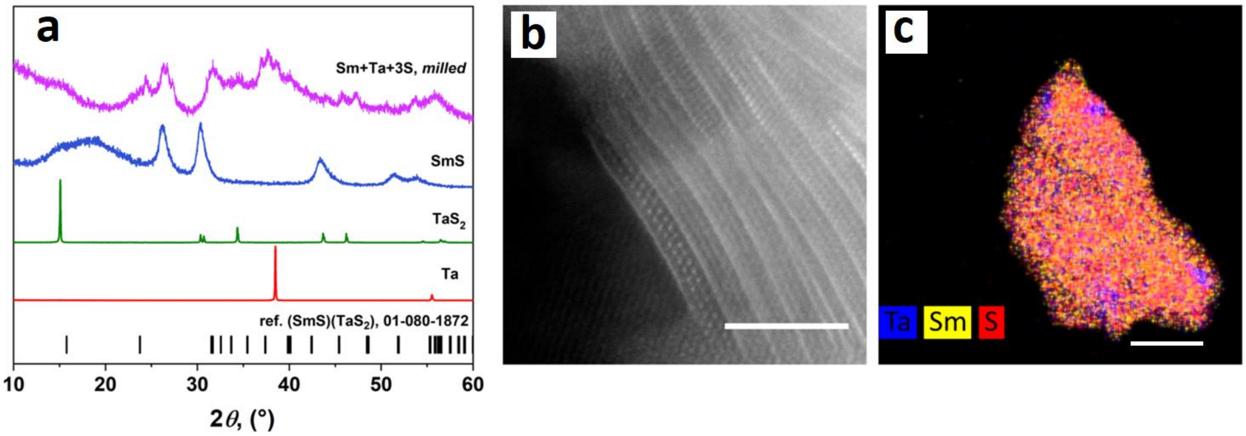

Supplementary Figure 5. Characterization of mechanochemically reacted Sm, Ta and S. a PXRD patterns, **b** HAADF-STEM image (scale bar, 5nm) and **c** EDS elemental mapping (scale bar, 100 nm) of the material produced by ball milling of Sm, Ta and S in the SPEX 8000M shaker mill for 12 hours.

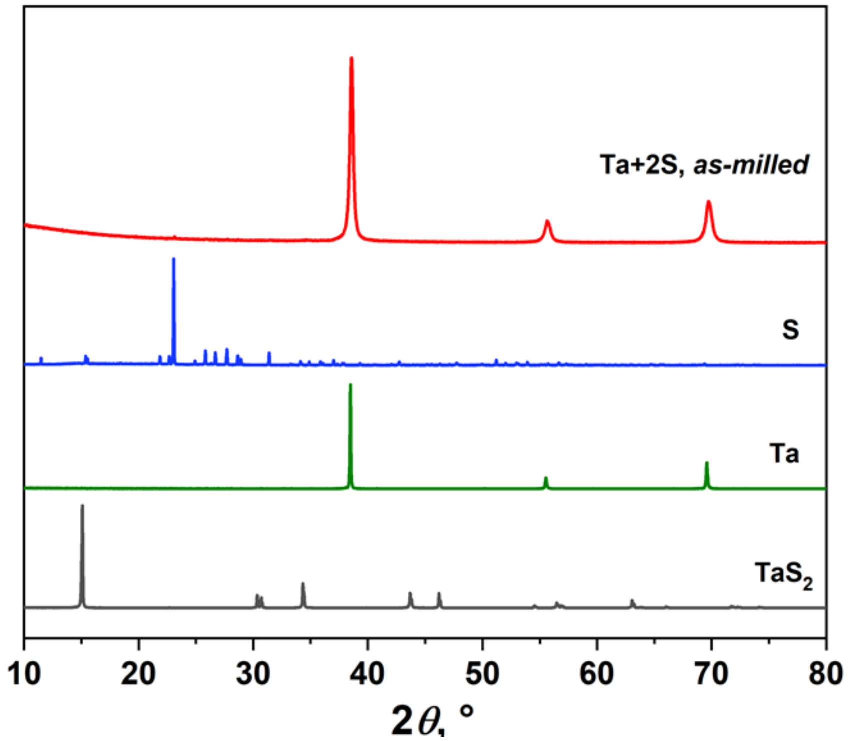

Supplementary Figure 6. Mechanochemical interaction between Ta and S. PXRD pattern of Ta and S taken in 1:2 molar ratio ball milled for 20 hours. PXRD patterns of TaS_2, Ta and S are shown as references.

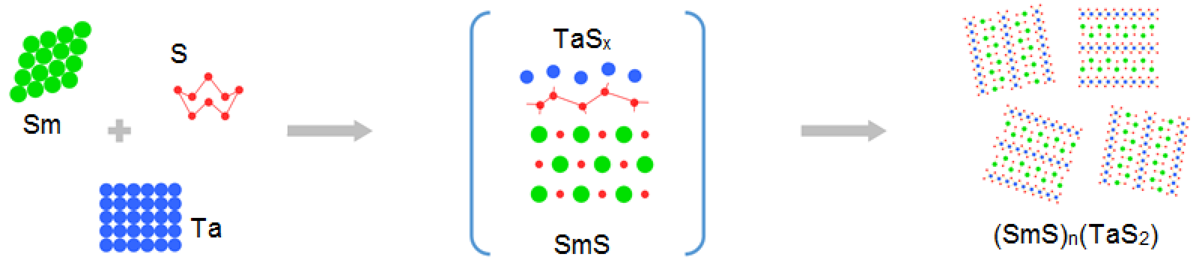

Supplementary Figure 7. Schematic presentation of the $(SmS)_n(TaS_2)$ heterostructure synthesis. Mechanochemical synthesis of $(SmS)_n(TaS_2)$ heterostructures from elemental Sm, Ta and S.

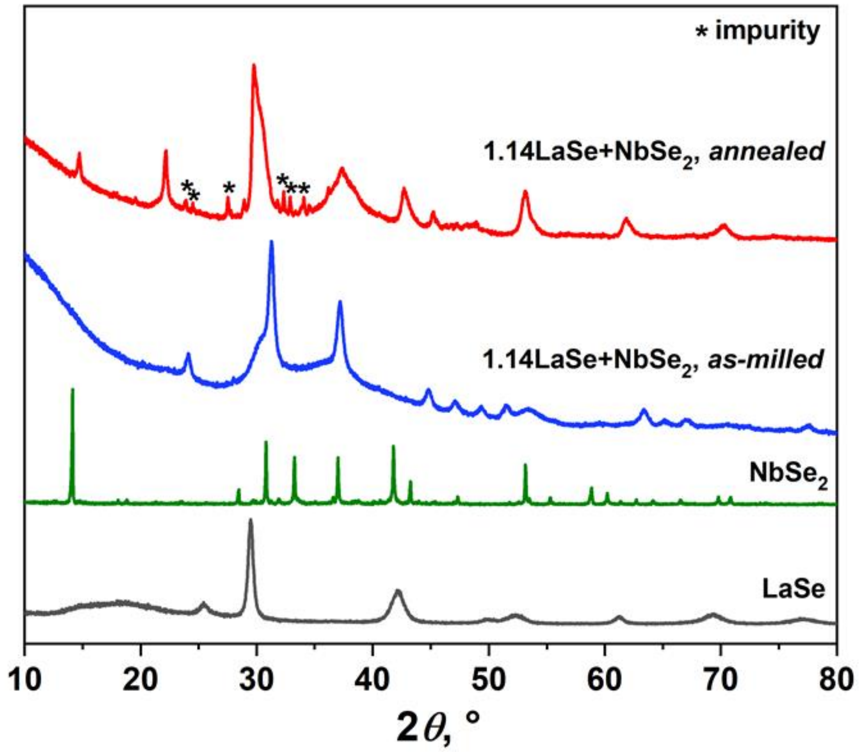

Supplementary Figure 8. PXRD patterns of the $(LaSe)_{1.14}(NbSe_2)$ heterostructure. PXRD patterns of $(LaSe)_{1.14}(NbSe_2)$ obtained after ball-milling of LaSe and $NbSe_2$ in a SPEX 8000 shaker mill for 20 hours, and annealing of the as-milled powder at 1000°C for 3 days. For reference, PXRD powder patterns of $NbSe_2$ and LaSe are presented. The peaks labeled as "impurity" probably belong to unidentified oxidized materials.

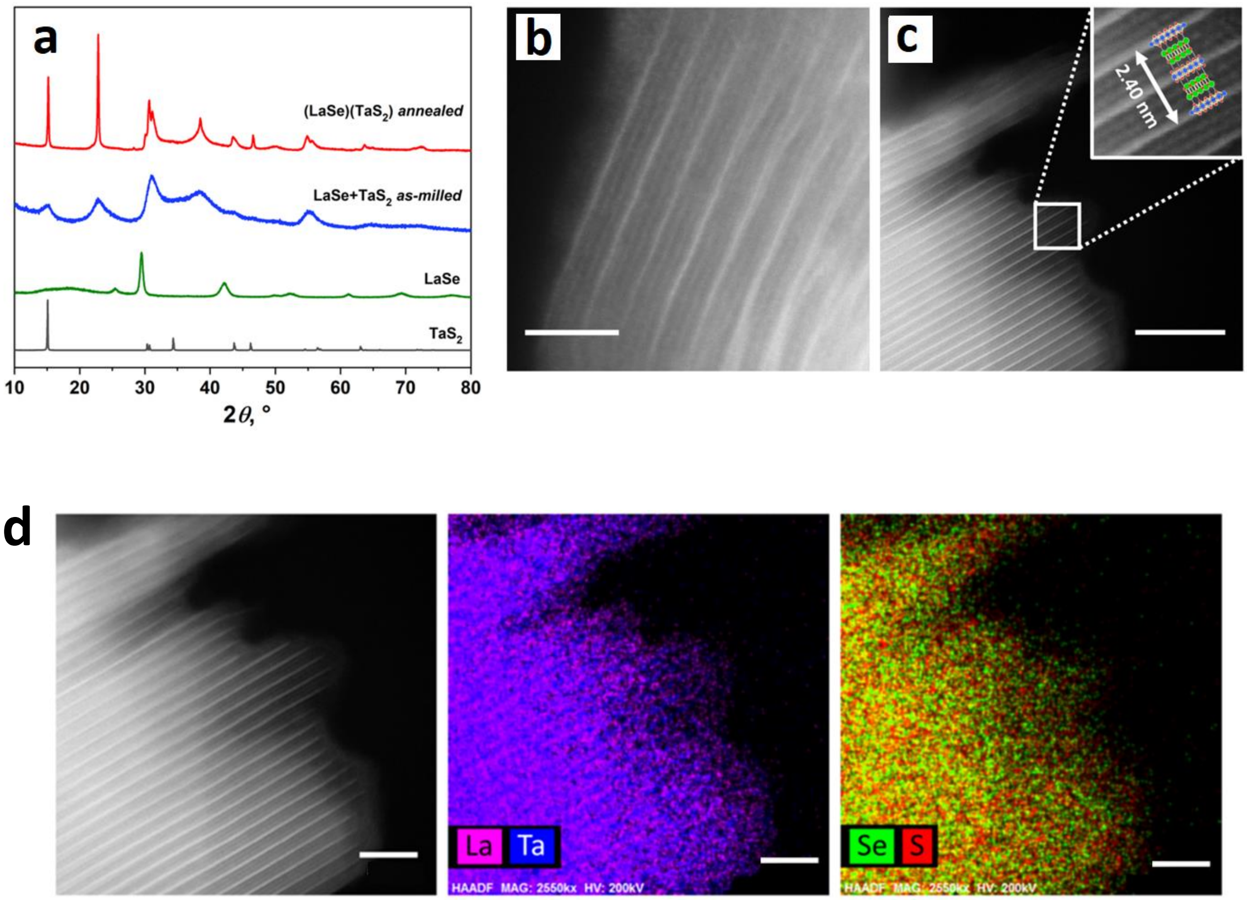

Supplementary Figure 9. Characterization of the (LaSe)(TaS_2) heterostructure. **a** PXRD patterns; **b** HAADF-STEM images of a sample after 30 hours of ball-milling LaSe and TaS_2 (scale bar 3 nm), and **c** subsequent annealing (LaSe)(TaS_2) formed at 1000°C for 3 days (scale bar 5 nm). **d** EDS elemental mapping of the annealed material (scale bars, 5 nm).

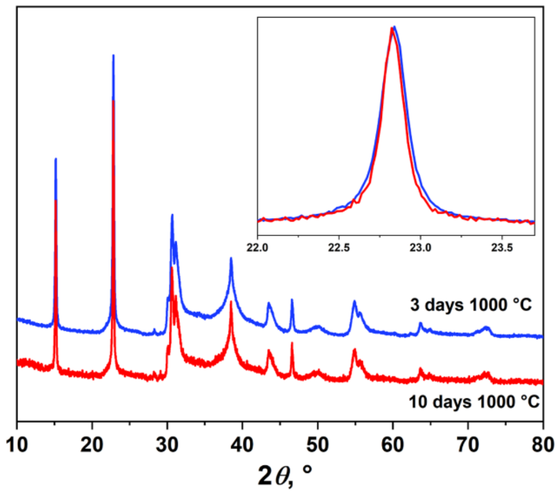

Supplementary Figure 10. PXRD patterns of annealed (LaSe)(TaS_2). PXRD patterns of (LaSe)(TaS_2) obtained after ball-milling of LaSe and TaS_2 in a planetary mill for 30 hours and annealing of the as-milled powder at 1000°C for 3 and 10 days. Inset compares the Bragg peak width for samples with different heat treatment times.

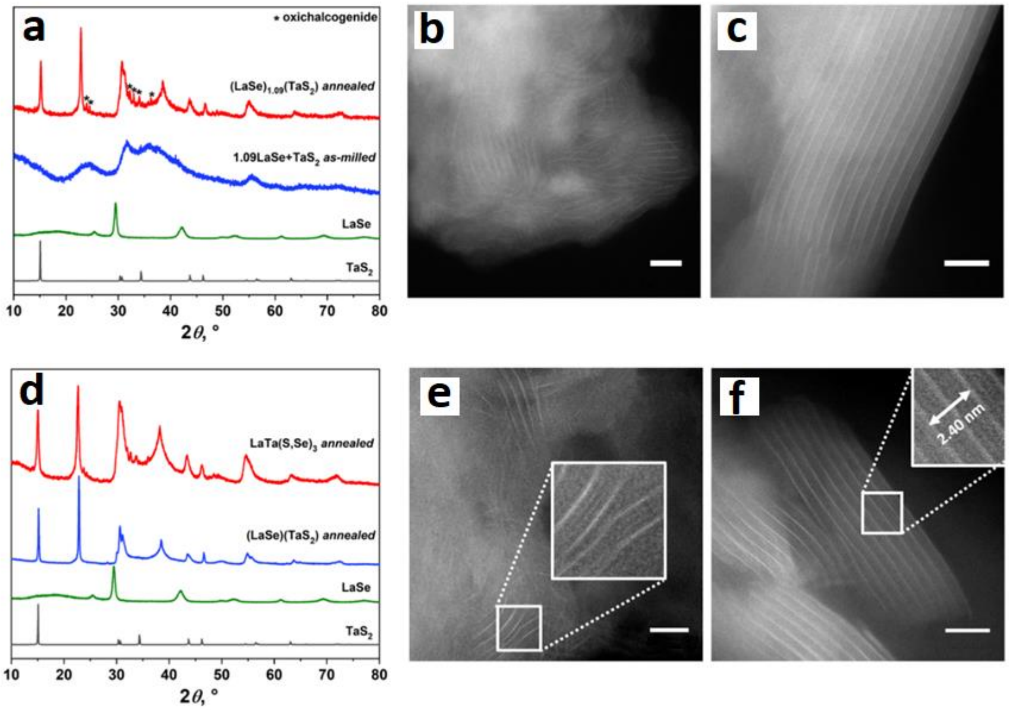

Supplementary Figure 11. Characterization of the $(LaSe)_{1.09}(TaS_2)$ heterostructure. **a** PXRD patterns of $(LaSe)_{1.09}(TaS_2)$ obtained after ball-milling of LaSe and TaS_2 in a SPEX 8000 shaker mill and after annealing at 1000°C for 3 days. **b** HAADF-STEM images of samples obtained after ball-milling and **c** annealing. **d** PXRD patterns and **e** and **f** HAADF-STEM images of a sample prepared from pure La, Ta, Se and S. The insets show enhanced atomic-scale views of the heterostructural arrangements of as-milled (in **e**) and annealed (in **f**) materials. Scale bars 5 nm.

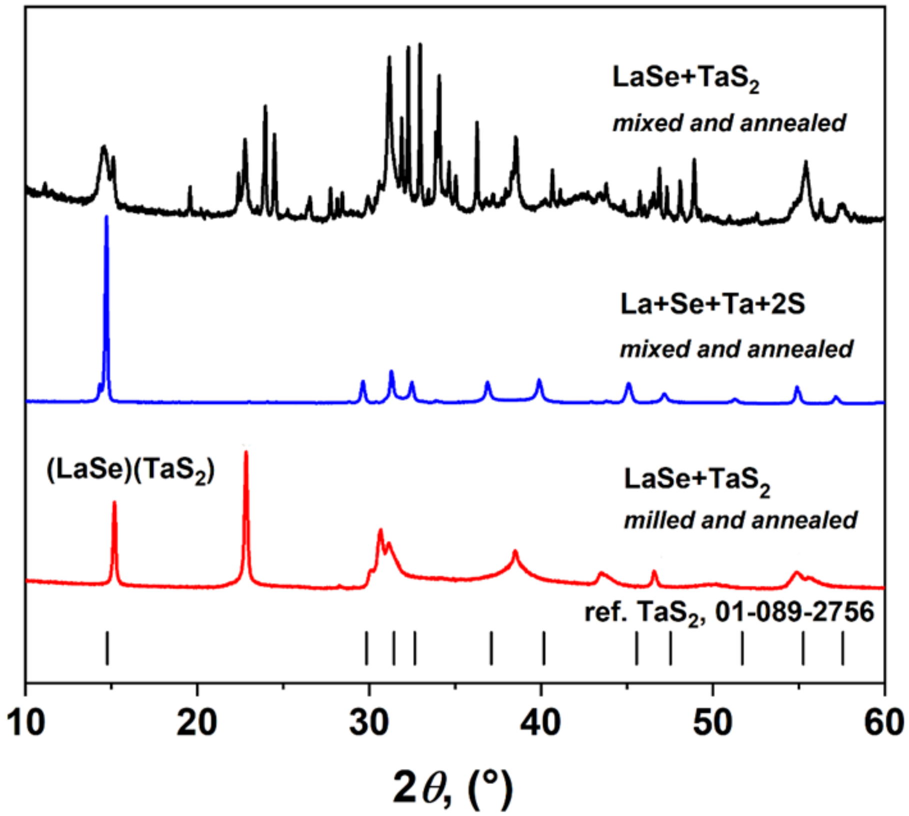

Supplementary Figure 12. PXRD patterns of annealed mechanical mixtures of Se, S, La and Ta, or LaSe and TaS_2. The elements of Se, S, La and Ta or binary metal chalcogenides, LaSe and TaS_2, were mixed in mortar in the appropriate ratios, sealed in quartz ampule under inert gas atmosphere, then heated for 3 days at 1000 °C. As a reference, the sample prepared by ball milling LaSe and TaS_2 with subsequent 3-day heat treatment is also provided. Vertical bars at the bottom of the chart correspond to Bragg peak positions of TaS_2.

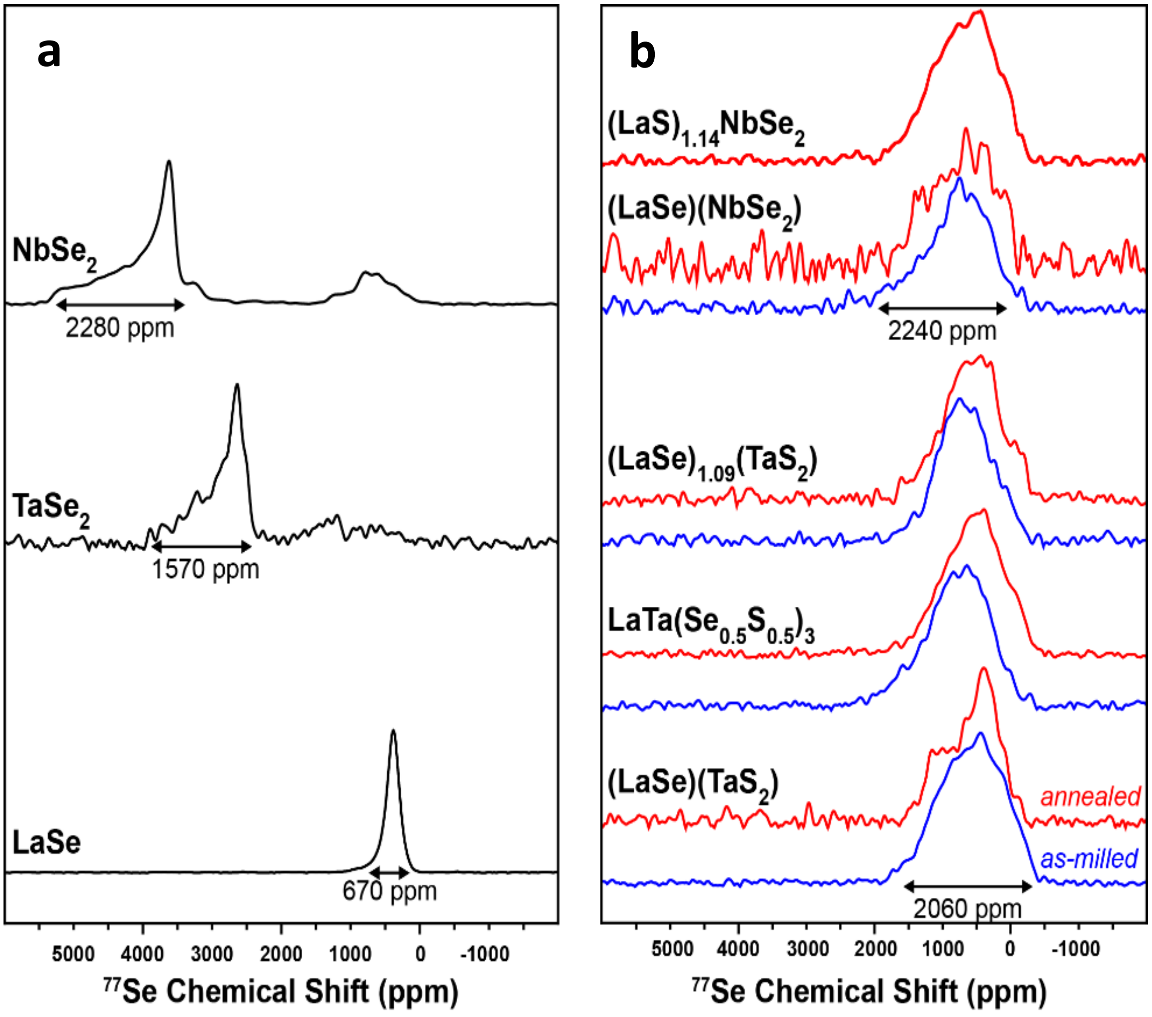

Supplementary Figure 13. SSNMR characterization of the $(REX)_{1+x}(MX_2)$ heterostructures. **a** Static ^{77}Se SSNMR spectra of LaSe, $NbSe_2$ and $TaSe_2$, and **b** $(REX)_{1+x}(MX_2)$ heterostructures. Spectra of as-milled and annealed samples are highlighted blue and red, respectively. Magic angle spinning (MAS) SSNMR experiments were attempted to try and observe isotropic ^{77}Se signals and determine to what extent anisotropic shifts and isotropic shift distributions contribute to the broadening of the static ^{77}Se NMR spectra. However, the sample of $NbSe_2$ needed to be highly diluted in polyfluoroethylene (ca. 5-10 wt% of heterostructure material) to eliminate bulk conductivity and permit MAS. Consequently, it was not possible to record MAS NMR spectra due to the low sensitivity brought about by the dilution. It was possible to perform MAS on the heterostructure material $(LaSe)(TaS_2)$ without dilution. The 25 kHz MAS NMR spectrum showed extensive spinning sidebands, confirming that shift anisotropy was responsible for broadening of the spectra. However, the isotropic and sideband peaks were very broad so that it was not possible to resolve isotropic signals for the different selenium environments.

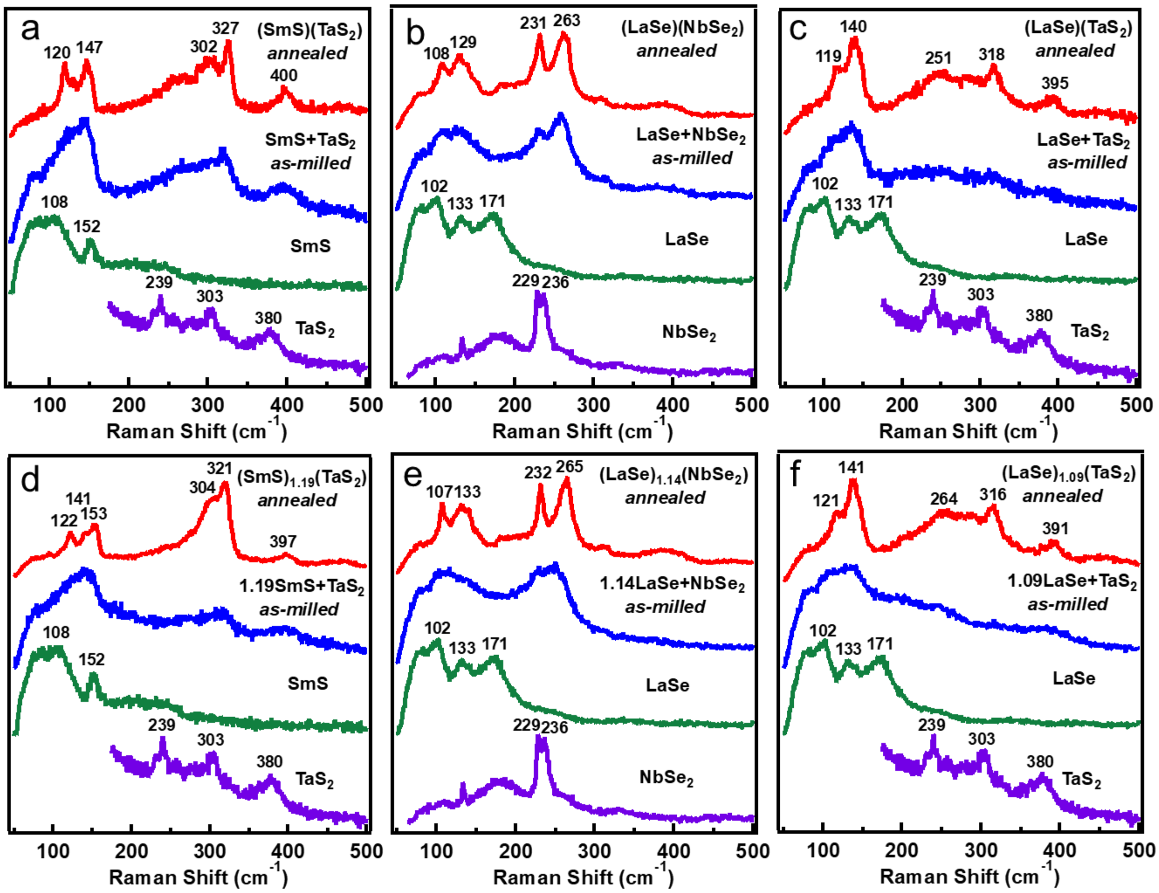

Supplementary Figure 14. Characterization of the heterostructures by Raman spectroscopy. Raman spectra of **a, d** $(SmS)_n(TaS_2)$, **b, e** $(LaSe)_n(NbSe_2)$ and **c, f** $(LaSe)_n(TaS_2)$ heterostructures; spectra of the precursors are shown as references.

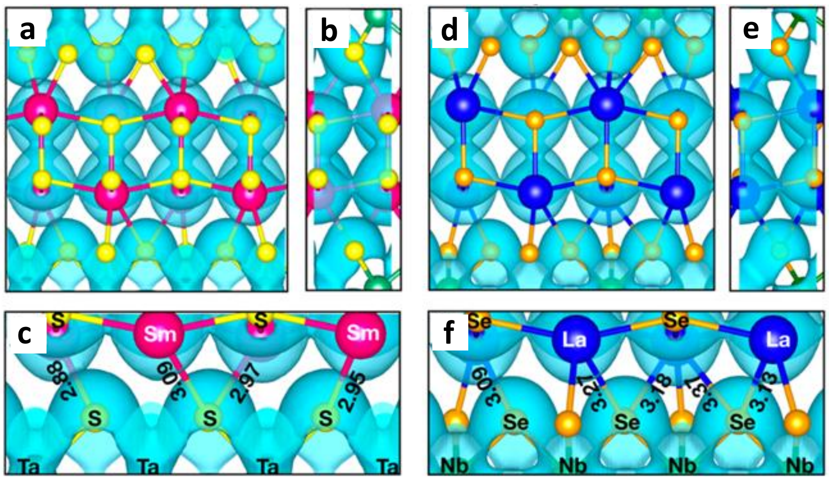

Supplementary Figure 15. Structural projections of $(REX)_n(MX_2)$ heterostructures from DOS calculations. Charge density plot for (left-panel) $(SmS)_{1.19}(TaS_2)$ and (right-panel) $(LaSe)_{1.14}(NbSe_2)$. **a, d** 001 projection; **b, e** 100 projection, intralayer charge sharing; **c, f** cut along 001 for better view of interlayer charge transfer (bond-lengths in Å). Isosurface value was set as 0.035 electron per $Å^3$.

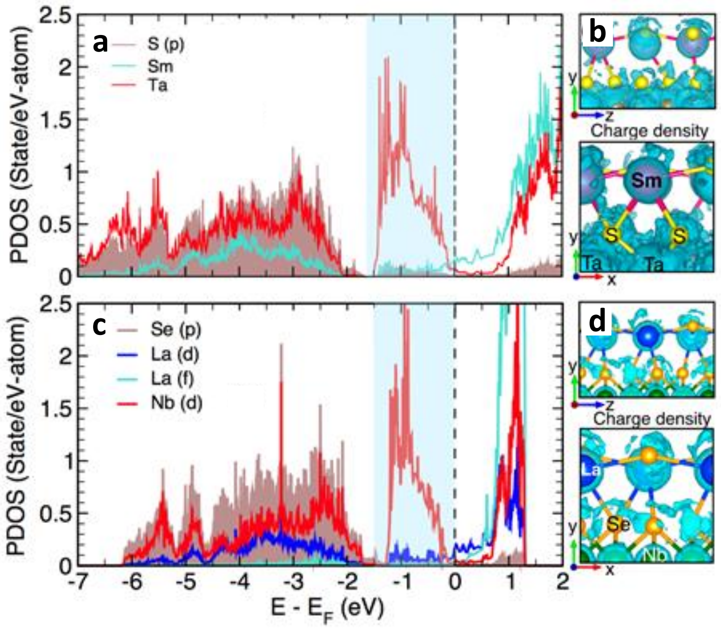

Supplementary Figure 16. DOS calculations for $(REX)_n(MX_2)$ heterostructures. Partial density of states and charge density plot for **a, b** $(SmS)_{1.19}(TaS_2)$ and **c, d** $(LaSe)_{1.14}(NbSe_2)$. The S (Se) in density of states plot is chosen from the interlayer boundary. Charge density plot for $(SmS)_{1.19}(TaS_2)$ in **b** and $(LaSe)_{1.14}(NbSe_2)$ in **d** for the energy range (-2.0 eV to Fermi-level) and (-1.5 eV to Fermi-level), respectively. The corresponding energies are highlighted in DOS.

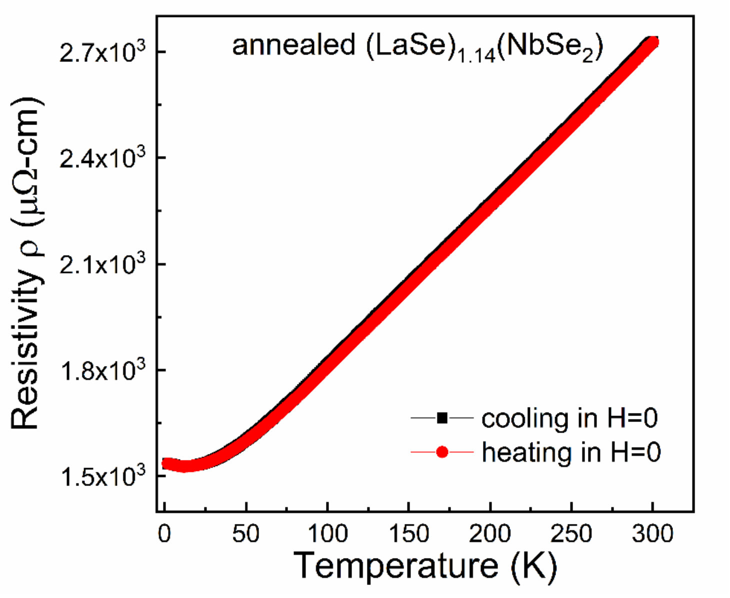

Supplementary Figure 17. Electrical resistivity characterization of the $(LaSe)_{1.14}(NbSe_2)$. Temperature dependence of electrical resistivity for annealed $(LaSe)_{1.14}(NbSe_2)$ in H = 0.

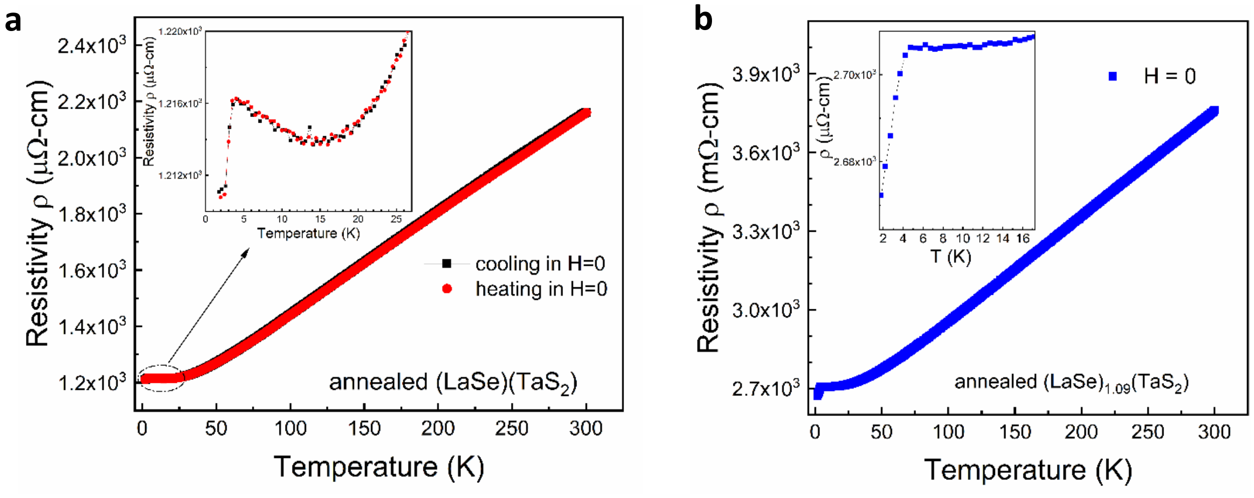

Supplementary Figure18. Electrical resistivity of the LaSe-TaSe$_2$ heterostructures. Temperature dependence of electrical resistivity for *annealed* (LaSe)$_n$(TaSe$_2$), **a** *n* = 1 and **b n =** 1.09 in H = 0.

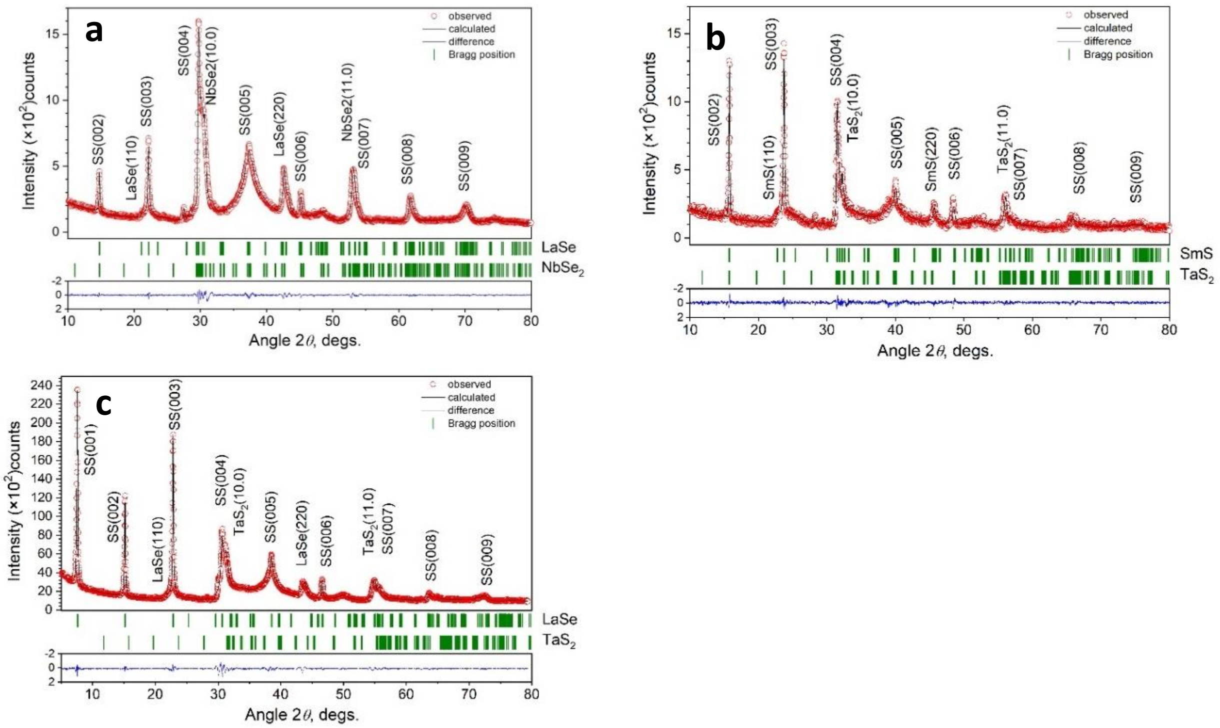

Supplementary Figure 19. **Structural refinement of misfit structures.** Results obtained using the Le Bail refinements of **a** $(LaSe)_n(NbSe_2)$, **b** $(SmS)_n(TaS_2)$, and **c** $(LaSe)_n(TaS_2)$ compounds. Vertical bars at the bottom of the plots indicate calculated positions of Bragg peaks from individual REX and MX_2 sublattices of misfit structures. Different orders of peaks associated with the LaSe/$NbSe_2$ (1:1) superstructure (SS) along the common *c*-axis are marked.

Supplementary Note 1: Density-Functional Theory (DFT) calculations

For theoretical calculations, we created large supercells consisting of seven MX_2 and four REX unit cells stacked along the *a*-axis to approximate the incommensurate crystal structures of $(REX)_n(MX_2)$, which after switching *b* and *c* can be described by space group symmetry *Ccc*2 with the corresponding (relaxed) unit cell dimensions listed in Supplementary Table 1 and atomic coordinates (also relaxed) listed in Supplementary Table 2.

For details on charge transfer in misfit compounds, we perform Bader charge analysis to determine partial charges on each site (Supplementary Table 3). For the start of charge self-consistency, we use total and atomic charges for $(SmS)_{1.19}(TaS_2)$: e^-(Ta) = 5; e^-(Sm) = 11; e^-(S) = 6; and $(LaSe)_{1.14}(NbSe_2)$: e^-(Nb) = 5; e^-(La) = 11; e^-(Se) = 6. We start with Ta (Nb) = 5, Sm = 11, and S = 6 electrons in the valence shell, but the charge distribution completely changes after self-consistency. For $(SmS)_{1.19}(TaS_2)$, the interlayer S [Z1-Z7] gain 1.5e^- while the intralyer gains 1.2e^- [Z9-Z11]. The Sm loses more than 1 electron (1.8e^-). Similarly, for $(LaSe)_{1.14}(NbSe_2)$, the interlayer Se [Z1-Z7] gain 0.8e^- while the intralyer gains 1.2e^- [Z9-Z11]. Here, La also loses nearly ~1.5 electrons (1.7e^-).

In Supplementary Figure 15, we plot charge density and project along (a) (001); (b) (100) – overlapping charge densities from Sm (red) and S (yellow) show stronger Sm-S intralayer coupling; (c) a zoomed interlayer region for $(SmS)_{1.19}(TaS_2)$ (left-panel). Similarly for $(LaSe)_{1.14}(NbSe_2)$ (d,e,f). We use isosurface level of 0.035 $e^-/Å^3$ in charge density plot.

As shown in Supplementary Figure 15, Sm-S and La-Se show variable interlayer bond-lengths with a minimum of 2.88 and 3.09 Å in $(SmS)_{1.19}(TaS_2)$ and $(LaSe)_{1.14}(NbSe_2)$, respectively. This further suggests that only few Sm-S (La-Se) play role in the stabilizing misfit compound through effective interlayer charge-transfer. The charge density due to S (Se) *lone-pairs* in TaS_2 ($NbSe_2$) layer points towards the Sm (La) atoms of the adjacent layer. This further indicates towards the cohesion of two slabs (e.g. between SmS and TaS_2, see Supplementary Figure 16) through charge transfer and is partially attributed to weaker covalent interactions. In order to maintain the necessary charge equilibrium, one electron has to be transferred from SmS (LaSe) slab to the TaS_2 ($NbSe_2$) slab to reach a stable electronic configuration (also see Supplementary Table 3).

Supplementary Table 1. Relaxed unit cell parameters of $(SmS)_{1.19}(TaS_2)$ and $(LaSe)_{1.14}(NbSe_2)$ materials.

System/Lattice-parameter	Space group	***a*** **(Å)**	***b*** **(Å)**	***c*** **(Å)**
$(SmS)_{1.19}(TaS_2)$	Ccc2 (#37)	22.809	22.624	5.773
$(LaSe)_{1.14}(NbSe_2)$	Ccc2 (#37)	24.191	24.107	6.075

Supplementary Table 2. Direct coordinates (x,y,z) in the relaxed unit cell of (Ta,Sm,S)/(Nb,La,Se) atoms used for the band-structure calculation of $(SmS)_{1.19}(TaS_2)$/$(LaSe)_{1.14}(NbSe_2)$ in space group of *Ccc*2 (#37).

XYZ_3	Wyckoff Position	(X,Y,Z) X=Ta; Y=Sm; Z=S	(X,Y,Z) X=Nb; Y=La; Z=Se
X1	4c	(0.25000, 0.25000, 0.56727)	(0.25000, 0.25000, 0.56736)
X2	8d	(0.03663, 0.24954, 0.06922)	(0.03710, 0.24913, 0.07021)
X3	8d	(0.60651, 0.24965, 0.06793)	(0.60610, 0.24938, 0.06805)
X4	8d	(0.17902, 0.25037, 0.07003)	(0.17919, 0.25086, 0.07198)
Y1	8d	(0.31214, 0.57388, 0.98707)	(0.31219, 0.57546, 0.98539)
Y2	8d	(0.56069, 0.57661, 0.00822)	(0.56098, 0.57727, 0.00674)
Y3	8d	(0.81341, 0.57839, 0.01643)	(0.81325, 0.57867, 0.01503)
Y4	8d	(0.06399, 0.57462, 0.99713)	(0.06355, 0.57587, 0.99554)
Z1	8d	(0.03381, 0.31982, 0.73740)	(0.03312, 0.31858, 0.74049)
Z2	8d	(0.17848, 0.31980, 0.73747)	(0.17827, 0.31879, 0.73661)
Z3	8d	(0.60932, 0.32113, 0.73727)	(0.60980, 0.32010, 0.73978)
Z4	8d	(0.46452, 0.32073, 0.73699)	(0.46497, 0.31971, 0.73593)
Z5	8d	(0.32214, 0.31910, 0.73747)	(0.32248, 0.31754, 0.74052)
Z6	8d	(0.89293, 0.32178, 0.73606)	(0.89265, 0.32082, 0.73567)
Z7	8d	(0.74876, 0.32199, 0.73625)	(0.74869, 0.32101, 0.73762)
Z8	8d	(0.31276, 0.54987, 0.49500)	(0.31274, 0.55045, 0.49121)
Z9	8d	(0.56283, 0.54842, 0.50095)	(0.56293, 0.54913, 0.50084)
Z10	8d	(0.81235, 0.54760, 0.50394)	(0.81231, 0.54842, 0.50505)
Z11	8d	(0.06201, 0.54950, 0.49825)	(0.06203, 0.55003, 0.49637)

Supplementary Table 3. Partial charges on inequivalent atoms in $(SmS)_{1.19}(TaS_2)$/$(LaSe)_{1.14}(NbSe_2)$ after Bader charge analysis. See Supplementary Table 2 for the explanation of X,Y and Z notations.

**XYZ_3**	**Wyckoff position**	**Partial charge $(SmS)_{1.19}(TaS_2)$**	**Partial charge $(LaSe)_{1.14}(NbSe_2)$**
X1	4c	4.786	4.8073
X2	8d	4.794	4.8107
X3	8d	4.786	4.7938
X4	8d	4.808	4.8039
Y1	8d	9.1917	9.3142
Y2	8d	9.1745	9.3226
Y3	8d	9.1825	9.3026
Y4	8d	9.1793	9.3204
Z1	8d	7.5846	6.8940
Z2	8d	7.5740	6.8795
Z3	8d	7.5367	6.9138
Z4	8d	7.4953	6.9040
Z5	8d	7.5879	6.8700
Z6	8d	7.4623	6.9001
Z7	8d	7.4507	6.9207
Z8	8d	7.2199	7.1506
Z9	8d	7.2602	7.1598
Z10	8d	7.2873	7.1832
Z11	8d	7.2501	7.1490
