# Supplementary material for: Unprecedented generation of 3D heterostructures by mechanochemical disassembly and re-ordering of incommensurate metal chalcogenides
Source: Nat Commun. 2020 Jun 12;11:3005. doi: 10.1038/s41467-020-16672-0 (PMC7293273; doi:10.1038/s41467-020-16672-0)
Supplement: Supplementary file 2 — Peer Review File [file 41467_2020_16672_MOESM2_ESM.docx]

Peer Review Information

**Journal:** Nature Communications

**Manuscript title:** Unprecedented Generation of 3D-Heterostructures by Mechanochemical Disassembly and Re-Ordering of Incommensurate Metal Chalcogenides

**Corresponding author name(s):** Dr Viktor Balema

**Editorial notes:**

**none**

**Reviewer comments & decisions:**

**Reviewer comments, first version:**

Reviewers' comments:

Reviewer #1 (Remarks to the Author):

Although the manuscript does describe intriguing formation of some new compounds by a combination of milling and annealing, the novelty is low as similar combinations of milling and annealing have been commonly used for making new alloys and compounds. In fact, milling alone is well known to induce phase transformation [e.g., Shen, et al., J. Mater. Chem. A 3(3285)2015; Ulbrich, et al., J. Magnet. Magnet. Mater. 493(165706)2020]. Further, combinations of milling in various solvents and post-milling annealing have also been recently used to demonstrate the possibility of manipulating phase/composition evolutions and magnetic properties of NdFeCoC [Tu, et al., Mater. Design 183(108140)2019].

The claim of taking “quantum interactions” to guide the development of a recipe for a new compound {abstract of the manuscript: The discovered solid-state processes are rooted in stochastic mechanochemical transformations guided by quantum interactions between chemically and structurally dissimilar solids toward atomic-scale ordering and offer a unique, so far unexplored alternative to conventional heterostructuring.} is scientifically improper, as “quantum interaction” is a term referring to physics in strongly correlated electronic systems [see, e.g., Dagotto, Science 309(257)2005.]

I opine that the manuscript is not suitable for Nature Communications, and that it should be revised and submitted to topical journals specializing in materials synthesis.
u


Reviewer #2 (Remarks to the Author):

The paper “Unprecedented Generation of 3D-Heterostructures by Mechanochemical Disassembly and Re-Ordering of Incommensurate Metal Chalcogenides” by Dolotko et al presents a systematic study of a novel, low cost way of generating van der Waals heterostructures between transition metal dichalcogenides and non-layered rare-earth metal monochalcogenides. Most interesting result of the paper is that simple ball milling of initial crystal or even elemental components leads to the formation of ordered heterostructures. Annealing of such heterostructures significantly increases its quality.
The paper is reasonably well written with a few exceptions. It provides a comprehensive analysis of the formation methods and characterisation by a variety of techniques. In general, I think it should be published in Nature Communications. Below are some consideration on how the paper can be improved.
1) The authors should provide experimental evidence of the formation mechanism of the van der Waals heterostructures. Ideally they need to stop the ball milling at intermediate stages and investigate the resulting products, which will give some insight into the mechanism of formation.
2) The theory of formation of (LaSe)nTaS2 is somewhat questionable. In the absence of definitive data, I would reduce the focus on this part, presenting it as suggestion. I agree that the NMR study of 77Se help in some way. The authors need to link the two parts better and provide stronger arguments towards the formation of (LaSe)nTaS2.
3) The extraction of the band-gap from the temperature dependence of the as-prepared samples in the temperature range of 200-300K is a bit naïve. In order to get a decent exponential fitting one need to have resistivity changing significantly, which is clearly not the case here. Most probably in the non-annealed samples there is a tunnelling barrier between crystallites formed by amorphous material, which leads to overall insulating behaviour.


Reviewer #3 (Remarks to the Author):

In the present paper Dolotko et al. report an original synthesis and preliminary characterization of a few misfit layered compounds (ML) of the form (REX)1+x(MX2) by combining mechanical milling with high temperature annealing. MLCs are made of a staking of distorted rocksalt slab and hexagonal transition metal dichalcogenides (TMDC) compound. MLC belong to the large class of the highly anisotropic 2D materials, but with a singular caveat that they are non-stoichiometric compounds with the a-axes of the two units being incommensurate. They are expected to reveal fascinating properties, like topological states and 2D superconductivity, especially if a single (REX)1+x(MX2) layer can be prepared and studied at low temperatures. In contrast to the more standard TMDC, which can be synthesized via many routes, MLC have been synthesized so far mostly via the chemical vapor transport (CVT) technique using halogens or halogen compounds as the transport agent. Obviously, CVT leaves halogen impurities in the MLC, which can greatly influence their physical properties, especially at low temperatures. In contrast to the TMDC, which are being explored intensively for a decade now, the research on MLC is rather limited, possibly due also to the great difficulties to synthesize them in single crystal form or as individual layers. The mechanochemical method for the MLC synthesis reported in this work is likely to open new vistas for their facile synthesis and the investigation of their properties. Therefore, I find this article of great interest and I recommend its publication in the present journal, although it may not have an immediate effect on the field of 2D materials.

I have nevertheless several comments on the ms. for consideration of the authors.

1. Technical issue: the equation 1+x=4a1/2a2 is not correct – it should be 1+x=4a2/2a1.

2. I feel that while the authors rightly cite some of the important historical papers on MLC, they ignore more recent work, in particular that of David Johnson.

3. The discussion of the “stoichiometric compounds” of the form (LnS)(TaS2) is not very clear. Since MLC are per definition non-stoichiometric- the (LnS)(TaS2) product must compensate the deviation from the known formula via defects; vacancies, antisites, etc. This point must be discussed in a more clear form. 4. Since MLC are very sensitive to oxidation, the secondary phase that the authors have indicated in their XRD could be associated with an oxide. I do not believe that the annealing at 1000 °C under helium atmosphere can provide sufficiently robust environment against partial MLC oxidation at such elevated temperatures.

5. I wonder if the authors have any electron diffraction data of their synthesized MLC, which could show if the layers suffer turbostratic-stacking disorder or not.

6. The authors hardly discuss the yield of the synthesis, other than stating of a secondary phase.

7. Did the authors try to exfoliate their MLC, perhaps the crystallites are too small for that purpose.

8. In the mixed S, Se MLC the authors started from a given binary precursors and they assume that there was no sulfur-selenium exchange in the two sublattices. This hypothesis should be verified, since 1000 °C for a few days can easily lead to S/Se exchange between the (REX) and TaS2 lattice. In principle, high resolution HAADF/STEM or EDS could address these issue. The SSNMR is of a limited value in this respect because, I believe that it is not sufficiently sensitive to minority phase.

9. I do not think I have seen the authors saying anything on the annealing at 1000 °C without millingdid they get any MLC under these conditions?

10. The authors should index the main XRD peaks in the diffraction patterns- at least once.

11. The Raman peaks are fairly broad which is indicative of a high degree of disorder in the crystallites. 12. Finally, I do not find myself in the DFT calculations. For example, the authors show three different numbers for the (SmS)1.19(TaS2): -7.3708; -1.5781 (Table 2) and -0.15 eV in Fig. 11. The significance of these numbers are really unclear to me.

In summary, I think the authors should address these points before the paper can be considered for publication in this journal.

**Author rebuttal, first version:**

**Reviewer #1**

“*Although the manuscript does describe intriguing formation of some new compounds by a combination of milling and annealing, the novelty is low as similar combinations of milling and annealing have been commonly used for making new alloys and compounds.*”

**Response:**

Our manuscript reports on *the discovery* of a unique low-temperature ordering of solid materials into heterostructured assemblies upon mechanical processing in the absence of solvents. We clearly demonstrate that *in contrast to common* *belief* about the destructive nature of mechanical milling, it *consistently and reproducibly* facilitates the formation of ordered 3D heteroatomic assemblies (heterostructures) from structurally dissimilar solids. Further, while mechanochemically generated misfit moieties *subsequently* order upon annealing, the thermal treatment is applied to the solids where heterostructured arrangements are *already formed*. Consequently, milling and heating are not interdependent steps, unlike conventional syntheses example that the reviewer appears to refer. Finally, milling in powder metallurgy is mostly done to prepare amorphous, metastable, or non-equilibrium alloys, whereas annealing drives such products to thermodynamic equilibrium and is undesired, [e.g. see C. Suryanarayana, *Mechanical Alloying and Milling*, Marcel Dekker, Inc., New York, NY, USA, 2004.; [Handbook of Non-Ferrous Metal Powders (Second Edition)](https://www.sciencedirect.com/book/9780081005439), Eds.: Neikov, O.D., Naboychenko, S.S., Yefimov, N.A.; Elsevier, Amsterdam (NL), Oxford (UK), Cambridge (MA, USA), 2019, 995 p.]

*“In fact, milling alone is well known to induce phase transformation [e.g., Shen, et al., J. Mater. Chem. A 3(3285)2015; Ulbrich, et al., J. Magnet. Magnet. Mater. 493(165706)2020]. Further, combinations of milling in various solvents and post-milling annealing have also been recently used to demonstrate the possibility of manipulating phase/composition evolutions and magnetic properties of NdFeCoC [Tu, et al., Mater. Design 183(108140)2019].”*

**Response:**

With all due respect to the reviewer’s opinion, none of the references presented are relevant.

- Tu, et al. [Mater. Design 183(108140)2019], report about the preparation of nanocrystalline materials by ball milling in organic solvents, while no such processes are utilized in our work.
- Shen, et al. [J. Mater. Chem. A 3(3285)2015] is concerned with mechanically generated allotropes of elemental phosphorus, while our paper deals with mechanochemical combining different types of binary compounds into multi-element heterostructured materials.
- Ulbrich, et al. [J. Mag. Magn. Mater. 493(165706)2020] report mechanochemical sulfurization of nickel that results in a binary Ni_3_S_2_ – a well-known sulfide that is a not a heterostructured material*.*

“*The claim of taking “quantum interactions” to guide the development of a recipe for a new compound {abstract of the manuscript: The discovered solid-state processes are rooted in stochastic mechanochemical transformations guided by quantum interactions between chemically and structurally dissimilar solids toward atomic-scale ordering and offer a unique, so far unexplored alternative to conventional heterostructuring.} is scientifically improper, as “quantum interaction” is a term referring to physics in strongly correlated electronic systems [see, e.g., Dagotto, Science 309(257)2005.]*”

**Response:**

Although the term “quantum interactions“ can be used in a broader sense then stated in the reviewer’s comment (e.g., see [R. K. Burdick](https://www.nature.com/articles/s41598-019-47651-1#auth-1) et al, “Modern Anesthetic Ethers Demonstrate Quantum Interactions with Entangled Photons**”** Scientific Reports  **9**,  11351, 2019), to remove pointed out ambiguity, we have modified the sentence in question to read: The discovered solid-state processes are rooted in stochastic mechanochemical transformations directed by electronic-structure-based (quantum) interaction between chemically and structurally dissimilar solids toward atomic-scale ordering and offer a unique, so far unexplored alternative to conventional heterostructuring.

*“I opine that the manuscript is not suitable for Nature Communications, and that it should be revised and submitted to topical journals specializing in materials synthesis.”*

**Response:**

We respectfully disagree with reviewer’s opinion for the reasons described above.

**Reviewer #2**

*“The authors should provide experimental evidence of the formation mechanism of the van der Waals heterostructures. Ideally, they need to stop the ball milling at intermediate stages and investigate the resulting products, which will give some insight into the mechanism of formation.”*

**Response:**

We performed the experiment suggested, whereby we ball milled LaSe with NbSe_2_ for 10, 20 and 30 hours in a planetary mill. The powders obtained were studied by PXRD and corresponding PXRD patterns have been added to the Supplementary Information file as Fig. S17. While diffraction data indicate a gradual disappearance of the Bragg peaks corresponding to the starting materials and appearance of those corresponding to the reaction product, the intermediates cannot be reliably identified there. Also, subsequent HAADF-STEM experiments did not allow for the reliable identification of long-lived reaction intermediates.

At the same time, the HAADF-STEM image of the *as-milled* SmS - TaS_2_ mixture shown in Fig. S3b (see the Supplementary Information file) clearly indicates the presence of exfoliated TaS_2_ nanosheets in the sample, which is in line with the reaction mechanism proposed in Fig. 2 (main text). A similar arrangement can be seen also in Fig. S8e in the Supplementary Information file. We enhanced the area of interest in Fig. S3b and added the enhanced fragment as inset to the revised figure. We have also added the following sentence in the main text: While both *as-milled* and *annealed* solids are similar to (SmS)(TaS_2_), still unreacted exfoliated TaS_2_ nanosheets are present in the as-milled sample (Fig. S3b, SI), which is in line with the reaction mechanism depicted in Fig. 2.

*“The theory of formation of (LaSe)nTaS2 is somewhat questionable. In the absence of definitive data, I would reduce the focus on this part, presenting it as a suggestion. I agree that the NMR study of 77Se help in some way. The authors need to link the two parts better and provide stronger arguments towards the formation of (LaSe)nTaS2.”*

**Response:**

The formation of (LaSe)nTaS2 follows same mechanism as other studied materials and we do not offer any theories specifically tailored to this system. Therefore, the reviewer’s comment is not justified given the available experimental results.

Unfortunately, STEM-EDS, Raman and solid-state NMR spectroscopies are unable to unambiguously confirm the exact locations of S and Se in the crystal lattice of (LaSe)_n_(TaS_2_) due to experimental limitations of these techniques. It turned out that ^77^Se NMR signals from the REX and TMX2 layers are too close to distinguish. HAADF/STEM and EDS experiments did not produce satisfactory resolution for either ball milled or annealed samples because of proximity of S and Se atoms in the lattice. Also, Raman spectra are not suitable for the identification of different atomic arrangements in this case. Therefore, we have to rely on the PXRD data. The lattice parameters of the *annealed* LaTa(Se_0.5_S_0.5_)_3_, especially its *a* parameter corresponding to the LaX sub-lattice, considerably deviate from those of (LaSe) _n_ (TaS_2_), **suggesting** different chalcogen occupancies in LaTa(Se_0.5_S_0.5_)_3_ and (LaSe)_n_(TaS_2_) heterostructures as stated in the manuscript. To make this statement even more suggestive, we replaced the word “can” in the original version by “may” in the revised manuscript.

We have also modified the following statement in the “Discussion” section: Clearly distinct from conventional heterostructuring techniques, the discovered synthetic approach enables direct synthesis of 3D heterostructures with site-separated metal-chalcogenide (S and Se) components that are inaccessible via conventional synthetic routes.

The revised version of the statement reads: Distinct from conventional heterostructuring techniques, the discovered synthetic approach may enable the 3D heterostructures with site-separated components that are inaccessible via conventional synthetic routes.

*“The extraction of the band-gap from the temperature dependence of the as-prepared samples in the temperature range of 200-300K is a bit naïve. In order to get a decent exponential fitting one needs to have resistivity changing significantly, which is clearly not the case here. Most probably in the non-annealed samples there is a tunnelling barrier between crystallites formed by amorphous material, which leads to overall insulating behavior.”*

**Response:**

We completely agree with the reviewer, and in the original version we explicitly stated the *estimated* nature of ΔE values. To account for other possibilities, we added the ones suggested by the reviewer: On the other hand, the observed phenomena may also be attributed to the presence of tunneling barriers between crystallites in *as-milled* samples that reduce their overall conductivity.

**Reviewer #3**

*“Technical issue: the equation 1+x=4a1/2a2 is not correct – it should be 1+x=4a2/2a1.”*

**Response:** The typo has been corrected.

*“I feel that while the authors rightly cite some of the important historical papers on MLC, they ignore more recent work, in particular that of David Johnson.”*

**Response:**

We apologize for the oversight, and have added the following references to the manuscript:

- Ref. 14. Grosse, C., Alemayehu, M., B., Mogilatenko, A., Chiatti, O., Johnson, D. C., Fischer, S.F. Superconducting Tin Selenide/Niobium Diselenide Ferecrystals. *Cryst. Res. Technol.* 52, 1700126 (2017)
- Ref. 18. Hamann, D. M., Hadland, E. C., Johnson, D.C. Heterostructures containing dichalcogenides-new materials with predictable nanoarchitectures and novel emergent properties. *Semicond. Sci. Technol.* **32**, 093004 (2017)

*“The discussion of the “stoichiometric compounds” of the form (LnS)(TaS2) is not very clear. Since MLC are per definition non-stoichiometric- the (LnS)(TaS2) product must compensate the deviation from the known formula via defects; vacancies, antisites, etc. This point must be discussed in a more clear form.”*

**Response:**

Although we did not use “stoichiometric compounds” directly (we used “stoichiometry(ies)” to reflect relative quantities of the elements forming misfit compounds), we agree and accept that this can be improved. Even though we already noted that defects (stacking faults) such as double/multiple TaS_2_ layers in (LnS)(TaS_2_) compensate for deviations from known/anticipated formulae, we have altered the relevant paragraph to further reiterate this point. In the revised version on the manuscript it reads: Detailed HAADF-STEM and EDS investigations (Fig. 4) of a favorably oriented particle of the *annealed* (SmS)(TaS_2_) reveal the presence of a TaS_2_–TaS_2_ double layer, suggesting that the excess of TaS_2_ used for its preparation is incorporated into the final product as adjacent TaS_2_–TaS_2_ layers, representing stacking faults. Similar structural arrangements, have been observed in other particles of the mechanochemically prepared (SmS)(TaS_2_) (Fig. S4, SI) and also reported in the literature for (BiS)(VS_2_)_n_ misfit materials (1 ≤ n ≤ 15). Besides stacking faults, vacancies, antisite and other defects may contribute to compositional irregularities in misfit compounds.

*“Since MLC are very sensitive to oxidation, the secondary phase that the authors have indicated in their XRD could be associated with an oxide. I do not believe that the annealing at 1000 °C under helium atmosphere can provide sufficiently robust environment against partial MLC oxidation at such elevated temperatures.”*

**Response:**

We included the following statement to address this comment: Further, the *annealed* (SmS)_1.19_(TaS_2_) contains a distinct secondary phase in its PXRD pattern. This minor, most likely samarium oxysulfide impurity, is not present in the starting materials or in the (SmS)(TaS_2_) product.

The presence of oxide phases has also been shown in the PXRD patterns where we could identify them.

*“I wonder if the authors have any electron diffraction data of their synthesized MLC, which could show if the layers suffer turbostratic-stacking disorder or not.”*

**Response:**

We could not obtain quality electron diffraction data to test this hypothesis because heterostructured crystallites in the MLC particles are misaligned. We are planning a separate collaborative research to address structural features of the mechanochemically produced MLCs that may be able to better address reviewer’s question.

*“The authors hardly discuss the yield of the synthesis, other than stating a secondary phase.”*

**Response**

We do not feel that “yield” is the right word to use in our case. It is usually associated with compositionally well-defined compounds that have been/can be isolated in a pure form. Using “conversion rate” is more appropriate, as now introduced in the following statements:

“...no residual precursors could be detected in this material before and after the annealing, which indicates their complete conversion into the reaction products.”

“Processing of an equimolar mixture of SmS and TaS_2_ in a planetary mill for 30 hours produces a powder, whose PXRD pattern (Fig. 3a) does not contain Bragg peaks of the starting materials indicating their complete conversion into the reaction products.”

“*Did the authors try to exfoliate their MLC, perhaps the crystallites are too small for that purpose.”*

**Response:**

Indeed, the crystallites are too small for the mechanical (Scotch tape) exfoliation. Exfoliation in organic solvents using ultrasound destroys complex MLC heterostructures due to cavitation and causes extensive oxidation. Thin film deposition approaches represent a more efficient way to obtain single and a few layers MLC materials. However, such experiments are outside of the scope of this manuscript.

*“In the mixed S, Se MLC the authors started from a given binary precursors and they assume that there was no sulfur-selenium exchange in the two sublattices. This hypothesis should be verified, since 1000 °C for a few days can easily lead to S/Se exchange between the (REX) and TaS2 lattice. In principle, high resolution HAADF/STEM or EDS could address these issue. The SSNMR is of a limited value in this respect because, I believe that it is not sufficiently sensitive to minority phase.”*

**Response:**

HAADF/STEM and EDS techniques did not produce satisfactory resolution for either ball milled or annealed samples because of proximity of S and Se atoms in the lattice. Therefore, we have no other choice but rely on indirect evidence. One of such evidence is the difference in the lattice parameters of the material prepared from the elements (La, S, Se and Ta) and MLCs obtained from the binary compounds (LaSe and TaS_2_). We stated the suggestive character of our conclusions in the original version of the manuscript and further emphasized them in the revised version as shown below:

The lattice parameters of the *annealed* LaTa(Se_0.5_S_0.5_)_3_, especially its *a* parameter corresponding to the LaX sub-lattice, considerably deviate from those of (LaSe)(TaS_2_), suggesting different chalcogen occupancies in LaTa(Se_0.5_S_0.5_)_3_ and (LaSe)_n_(TaS_2_) heterostructures, which may be explained by assuming the preferential location of Se in the LaX sub-lattice of (LaSe)_n_(TaS_2_).

We have also modified the relevant statement in the Discussion section. Now it reads: Distinct from conventional heterostructuring techniques, the discovered synthetic approach may enable the 3D heterostructures with site-separated components that are inaccessible via conventional synthetic routes.

*“I do not think I have seen the authors saying anything on the annealing at 1000 °C without milling did they get any MLC under these conditions?”*

**Response:**

We performed the requested experiments and annealed mechanic (non-milled) mixtures of S, Se, Ta and La, as well as LaSe and TaS_2_ at 1000 ^o^C for 3 days. The PXRD patterns of the materials formed have been added to Supplementary Information as Fig. S18. In both cases, materials identical to the ones prepared upon mechanical milling and subsequent annealing did not form.

The following statement was added to the main text of the revised manuscript. Remarkably, mixing of La, Ta, S and Se in the 1:1:1.5:1.5 proportion and heating them without milling at 1000^o^C for 3 days does not produce LaTa(Se_0.5_S_0.5_)_3_ (Fig. S18), thus confirming the critical role of milling in the synthetic process.

*“The authors should index the main XRD peaks in the diffraction patterns - at least once.”*

**Response:** Done in Fig. S9.

*“The Raman peaks are fairly broad which is indicative of a high degree of disorder in the crystallites.”*

**Response:**

We respectfully disagree with the reviewer's statement. The Raman peak shape (width) is determined by many intrinsic properties of the material. Raman spectra of compounds that have similar composition, but different crystallinities can be different. The degree of the disorder could be one of the reasons that may explain the broadening of the Raman peak, but there are other factors such as crystal size, morphology and local environment which can vary for different samples and may cause the broadening of the Raman peaks. Also, the Y-axis scale in Fig. 10 may have led one to believe the peaks are wider than they actually are. We have updated Fig. 10 to eliminate the visual ‘broadening’ effect.

*“Finally, I do not find myself in the DFT calculations. For example, the authors show three different numbers for the (SmS)1.19(TaS2): -7.3708; -1.5781 (Table 2) and -0.15 eV in Fig. 11. The significance of these numbers are really unclear to me.”*

**Response:**

To clarify these calculated values of Total Energy and Formation Energy, we have added in an explanation of total energy and formation energy within the text before those number appear. In addition, we have added an extra column in Table 2 for the energy ($\Delta E)$ given relative to the 2H-MX_2_ compounds, which explicitly shows the relative stability of the heterostructure formation, which is what is plotted in Fig. 11.

As stated in the text, “E_form_ is the total energy relative to the weighted probabilities of the constituent-elemental energies; it is the energy gained (or lost) in formation of the heterostructure. Whereas, E_tot_ is the energy of the heterostructure relative to atomic vacuum.” The relative energies $\Delta E$ are plotted in Fig. 11, as is also now clarified in the caption.

For (SmS)_1.19_(TaS_2_) the -7.3708 and -1.5781 eV/atom in the table are total-energy (E_total_) and formation energy (E_form_), respectively. The total energy is relative to atomic vacuum, whereas formation energy reveals the energy “gain (or loss)” when forming the heterostructure from the constituent-element ground state structures, which is measure in calorimetry, for example.

The value of ~(-0.15) eV shown in Fig. 11 (left-panel) reflects the difference of total energy per atom for (SmS)_1.19_(TaS_2_) compared to 2H-TaS2.

We updated the captions in Fig. 11 and Table 2 accordingly.

**Reviewer comments, second version:**

REVIEWERS' COMMENTS:

Reviewer #2 (Remarks to the Author):

The authors satisfactory responded to two of my comments. I would still suggest the authors to review the estimation of the activation energy. First of all, the authors do not explain the origin of the activation energy (band gap? Schottky barrier?). Second, I insist that the measured temperature range is not sufficient to apply the exponential fit.
For the rest the manuscript has improved and, in principle I support the publication in Nature Communications.


Reviewer #3 (Remarks to the Author):

The revised version addressed most of my concerns and I am happy to recommend this ms. for publication in the journal. In particular, I am very glad that the author did several important control experiments in which the samples were mechanically mixed and annealed without the long milling process. These control experiments reinforce their proposed growth mechanism, i.e. that the TaS2 has to be exfoliated and then mate with the SmS layers formed chemically. The new control experiments with 10, 20 and 30 h annealing, which shed further light on the growth mechanism of the MLC. I am also happy to see the NMR data which confirms that the TaS2 is present in trigonal biprism coordination and not in the octahedral (1T) polytype. I am also happy to see that the theoretical calculations have been written in more clearly and can be followed much more easily now. I regret that the quality of the product is far less than that obtained via CVT, which excludes high resolution STEM-EELS analysis with atomic precision. It also excludes the possibility to carry out the highly informative electron diffraction analysis, which could shed light on the turbostratic relationship between the layers. Should they had higher quality MLC, I would recommend them to try exfoliating them mechanically or mechanochemically. I am glad that the authors clarified the conversion rate of their product and that they agree that some oxysulfides are unavoidable under the present annealing conditions. Should they add little bit of hydrogen gas or CO, they could possibly get rid of this minority phase.

I think that the quality of the paper and the important results obtained in this study merit its publication in the present journal and I am happy to recommend this paper for publication.

**Author rebuttal, second version:**

Reviewer #2:  *I would still suggest the authors to review the estimation of the activation energy. First of all, the authors do not explain the origin of the activation energy (band gap? Schottky barrier?). Second, I insist that the measured temperature range is not sufficient to apply the exponential fit.*

Response: we have removed the following paragraph from main text

The ρ(T) relationship in the temperature range between 200 and 300 K can be described by the Arrhenius relation, R(T) ~ exp(ΔE/k_B_T), where ΔE is the activation energy of the conduction process, k_B_ is the Boltzmann constant and T is the temperature. The values of ΔE estimated from the *lnρ* vs *1/T* linear fits (insets in Fig. 13a and 13b) attain 6.7 meV and 15.8 meV for *as-milled* (SmS)(TaS_2_) and (LaSe)(NbSe_2_), respectively, which is close to the values reported for heterostructured semiconductors, such as boron-nitride/graphene for example. On the other hand, the observed phenomena may also be attributed to the presence of tunneling barriers between crystallites in *as-milled* samples that reduce their overall conductivity.

We also removed insets from Fig. 8.

**Reviewer comments, third version:**

**Author rebuttal, third version:**
